# MdMYB52 regulates lignin biosynthesis upon the suberization process in apple

**DOI:** 10.3389/fpls.2022.1039014

**Published:** 2022-10-06

**Authors:** Xuan Xu, Gea Guerriero, Roberto Berni, Kjell Sergeant, Cedric Guignard, Audrey Lenouvel, Jean-Francois Hausman, Sylvain Legay

**Affiliations:** Luxembourg Institute of Science and Technology, Esch/Alzette, Luxembourg

**Keywords:** russeting, suberin, *malus* x *domestica*, *nicotiana benthamiana*, MYB-family transcription factor, MdMYB52, transcriptomics, lignin

## Abstract

Our previous studies, comparing russeted vs. waxy apple skin, highlighted a MYeloBlastosys (Myb) transcription factor (MdMYB52), which displayed a correlation with genes associated to the suberization process. The present article aims to assess its role and function in the suberization process. Phylogenetic analyses and research against *Arabidopsis thaliana* MYBs database were first performed and the tissue specific expression of MdMYB52 was investigated using RT-qPCR. The function of MdMYB52 was further investigated using Agrobacterium-mediated transient overexpression in *Nicotiana benthamiana* leaves. An RNA-Seq analysis was performed to highlight differentially regulated genes in response MdMYB52. Transcriptomic data were supported by analytical chemistry and microscopy. A massive decreased expression of photosynthetic and primary metabolism pathways was observed with a concomitant increased expression of genes associated with phenylpropanoid and lignin biosynthesis, cell wall modification and senescence. Interestingly key genes involved in the synthesis of suberin phenolic components were observed. The analytical chemistry displayed a strong increase in the lignin content in the cell walls during MdMYB52 expression. More specifically, an enrichment in G-Unit lignin residues was observed, supporting transcriptomic data as well as previous work describing the suberin phenolic domain as a G-unit enriched lignin-like polymer. The time-course qPCR analysis revealed that the observed stress response, might be explain by this lignin biosynthesis and by a possible programmed senescence triggered by MdMYB52. The present work supports a crucial regulatory role for MdMYB52 in the biosynthesis of the suberin phenolic domain and possibly in the fate of suberized cells in russeted apple skins.

## Introduction

Russeting is considered as a major fruit surface defect in apple, leading to price downgrading (Skene, 1982). In fruits, this rough and brown phenotype is mainly due to an accumulation of suberin in the walls of hypodermal cells. Suberized cells finally die and constitute this brown layer on the fruit surface (Faust and Shear, 1972). Suberin is an insoluble heteropolymer for which the structure is still under debate (Bernards, 2002; Graça, 2015; Vishwanath et al., 2015; Woolfson et al., 2022). Previous studies defined the suberization process by the synthesis of two distinct polyaliphatic and polyphenolic structures. The polyaliphatic component is considered as a glycerol-based polyester polymer containing embedded specific waxes such as alkyl-hydroxycinnamates (Bernards, 2002; Franke and Schreiber, 2007; Domergue and Kosma, 2017). This domain has been widely studied these last decades and displays a monomer composition mostly represented by high amount of bi-functional oxygenated fatty acids including α,ω-dicarboxylic acids and ω-hydroxyacids, which are esterified with glycerol and ferulic acid units forming the reticular structure. The structure and composition of polyaromatics found in suberin is less clear. Whereas there is a consensus about a probable synthesis of a polyaromatic polymer in the primary cell wall during the suberization process, the occurrence and composition of some polyaromatic structures in between aliphatic layers is still under debate (Bernards, 2002; Graça, 2015; Woolfson et al., 2022). Compared to lignified cell walls, it seems that suberized tissues contain a significantly higher amount of hydroxycinnamic acids (mainly ferulic acid) whereas the monolignol content is reduced. This has conducted to the hypothesis that this polyaromatic polymer is not exactly lignin (Bernards, 2002). However, other studies conducted from cork and native potato periderms showed that suberin-associated polyaromatics are highly similar to a lignin enriched in guaiacyl units (Pascoal Neto et al., 1995; Marques and Pereira, 2013; Graça, 2015).

The suberin biosynthesis pathway is mostly unraveled. It requires a tight interplay between multiple metabolic pathways including glycerol biosynthesis, cell wall modification, phenylpropanoid and lipid biosynthesis (Graça, 2015; Legay et al., 2015; Vishwanath et al., 2015; Woolfson et al., 2022). The understanding of the suberin biosynthesis regulation has also been widely improved this last decade. A significant number of MYeloBlastosys (Myb) family transcription factors were identified as suberin master regulator, which were able to regulate all the different above-cited metabolic pathways i.e. cell wall remodelling, lignin and lipidic building blocks biosynthesis and assembly. In Arabidopsis, this includes MYB41, MYB53, MYB92, MYB93, MYB9, MYB102, SUBERMAN, whereas in apple only MdMYB93 has been described so far (Kosma et al., 2014; Lashbrooke et al., 2016; Legay et al., 2016; Cohen et al., 2020; Shukla et al., 2021). Nevertheless, multiple orthologous genes of the above-cited Arabidopsis suberin regulatory MYB transcription factors displayed an increased expression in russeted apple fruit skin, suggesting that these might be involved in the suberization process in apple as well (Legay et al., 2015; André et al., 2022). Using our transcriptomic datasets, we also identified number of other characterized and uncharacterized MYB transcription factors. Among these, we selected MD05G1011100 (Daccord et al., 2017) or MDP0000852158 (Velasco et al., 2010), which is similar to the Arabidopsis MYB52 transcription factor and displayed a significant increased expression in russeted (suberized) apple fruit skin (Legay et al., 2015; Legay et al., 2017; André et al., 2022). In a previous work, we showed that MD05G1011100 is able to positively regulate the expression of MdOSC5, an oxidosqualene cyclase, which is involved in the synthesis of the lupan series of pentacyclic triterpenes, the latter being specifically accumulated in suberized fruit skin (Falginella et al., 2021). In *A. thaliana*, MYB52 has been descried as a negative regulator of pectin demethylesterification in seed coat mucilage (Shi et al., 2018). MYB52 only slightly induces the expression of genes involved in the secondary cell biosynthesis pathway and also indirectly causes a significant decrease in secondary cell wall thickening in fibres (Zhong et al., 2008). Interestingly, our previous transcriptomic work showed that a massive cell wall remodelling and lignification process occur during the suberization process in apple fruit skin. This led us to hypothesize that MD05G1011100/MdMYB52 might have a role in the regulation of the cell wall component such as hemicellulose or lignin during the suberization process. To validate this hypothesis, a transient expression of MD05G1011100 was performed in *N. benthamiana* leaves. Leaf samples were further analysed using an integrative approach combining whole gene expression profiling study, analytical chemistry and microscopy.

## Materials and methods

### Plant material

For gene isolation and qPCR analysis, the *Malus* x *domestica* ‘Cox Orange Pippin (COX-B)’, ‘AG94’, ‘Golden delicious’, ‘Court Pendu Gris (CPG)’, ‘Jonagold’, ‘Patte de loup’ samples were collected from three trees (biological replicates) from the orchard of the Walloon Agronomic Research Center of Gembloux (CRA-W) in 2013 (Legay et al., 2015; Legay et al., 2017). Shortly, from each tree, 10 apples were sampled from the south side of the tree at a height ranging from 1.20 to 2.20 m. An additional sampling has been performed for ‘Cox Orange Pippin’ variety (COX-L) from a private orchard in Luxembourg in 2013. Finally, samples collected during the fruit growth kinetic of the semi-russeted ‘Reinette du Canada Blanc’ and heavily russeted ‘Reinette du Canada Grise’ varieties described in André et al. (2022) were also used. Finally, Cox Orange Pippin, the russeted and waxy patches (areas) were carefully excised with scalpels taking care to remove the flesh as much as possible. The resulting exocarp samples were directly flash-frozen in liquid nitrogen and stored at -80°C until RNA extraction.

### Phylogenetic analysis

A phylogenetic analysis comparing the *M. x domestica* MD05G1011100-MdMYB52 to the Arabidopsis thaliana MYB transcription factor set, previously used in Legay et al. (2016), was performed using three different algorithm, namely MUSCLE (www.ebi.ac.uk/Tools/msa/muscle/), Clustal Omega (www.ebi.ac.uk/Tools/msa/clustalo/) and NCBI Cobalt (https://www.ncbi.nlm.nih.gov/tools/cobalt/re_cobalt.cgi). Trees were generated using IQ-TREE (http://iqtree.cibiv.univie.ac.at/?user=guest&jobid=220909113149) and visualized using the ITOL online tool (https://itol.embl.de/).

### RNA extraction, reverse transcription and qPCR

For the gene isolation, total RNA extraction was performed from the semi-russeted ‘Cox Orange Pippin’ variety using an adapted CTAB buffer-based extraction protocol (Gasic et al., 2004). For *N. benthamiana*, total RNA were extracted from two groups (p103::MD05G1011100/pBIN61-p19 (overexpression) and P103 (empty vector)/pBIN61-p19 (control)) of agroinfiltrated leaves. Three and 4 biological replicates were used for RT-qPCR and RNA-Seq, respectively. Each biological replicate was comprised of a pool of 3 plants with 4 agroinfiltrated leaves per plant. Total RNA extracts were obtained using the RNeasy plant mini kit (QIAGEN, Leusden, The Netherlands) coupled with on-column DNase I treatment, following the manufacturer’s guidelines. Total RNA integrity (RIN>8) and purity were assessed using a 2100 Bioanalyzer (Agilent Technologies, Santa Clara, CA, USA) and a Nanodrop ND1000 spectrophotometer (Thermo scientific, Villebon-sur-Yvette, France), respectively. Total RNA was quantified using a Qubit RNA assay kit (Life technologies, Carlsbad, CA, USA). For gene isolation and RT-qPCR, reverse transcription was carried out as described in our previous work (Legay et al., 2015). Data were analyzed using Biogazelle qbase+ v3.3. The geNorm analysis recommended to use 4 reference genes (EF1, SAND, UK, PP2A) to properly normalize the expression data (**Supplementary Table 1**). A statistical analysis has been performed for each gene and each dataset independently in SPSS v17 using log base 2 transformed data as input and an ANOVA followed by a Tukey *Post Hoc* test.

### Gene isolation and cloning

A cDNA library generated from the total RNA extracted from *M.* x *domestica* cv. “Cox Orange Pippin” was used to isolate the MD05G1011100 contig using the following primers: Forward(F) 5’- CACCATGGAGGATTATGGGGATGA-3’ and Reverse(R) 5’- AGAAGAGATCCCCACACCAA -3’. Amplification was carried out using the Q5 High Fidelity 2X Master Mix (New England Biolabs, Ipswich, MA, USA) following the manufacturer’s instructions (Tm=65°C). The PCR product was purified using the QIAquick PCR purification Kit (QIAGEN), cloned into pENTR/D TOPO and further used to transform *E. coli* One Shot TOP10 competent cells, which were grown on LB/kanamycin (50 mg/L) plate according to the manufacturer’s guidelines (Thermo scientific). The plasmid was then extracted using the QIAprep Spin Miniprep Kit (QIAGEN, Leusden, The Netherlands) and the insert was sequenced. The LR clonase cloning protocol (Thermo scientific) was used to recombine the insert into pEarleyGate103(p103) vector (Earley et al., 2006) to obtain a MD05G1011100-GFP fusion driven by the CaMV35S promoter. After quality check, P103::MD05G1011100 was finally transformed into *Agrobacterium tumefaciens* GV3101-pMP90.

### Infiltration by *Agrobacterium tumefaciens*

Three *A. tumefaciens* GV3101-pMP90 strains (p103::MD05G1011100, p103-empty and pBIN61-p19) were grown in 50 mL of LB liquid medium supplemented with gentamycin (30 mg/L), rifampicin (10 mg/L), kanamycin (50 mg/L), and acetosyringone (30 mg/L). As *A. tumefaciens* is not sensitive to the CcdB protein, we used the p103-empty vector as control in the present work (Traore and Zhao, 2011).

The cultures in 100 mL Erlenmeyer flasks were agitated at 130 rpm/28°C for 48 h. After centrifugation (1000 g/10 min), cultures were re-suspended in infiltration buffer (20 mM MES, 20 mM MgSO_4,_ 150 mg/L acetosyringone). After 3 hours, p103::MD05G1011100, p103-empty and pBIN61-p19 *A. tumefaciens* cultures were mixed and adjusted to OD_600 =_ 0.8, 0.8 and 1, respectively.

Three independent transient expression experiments have been performed for the RNA-Seq, qPCR validation and the analytical chemistry using four-weeks old *N. benthamiana* plants. Each biological replicate was constituted by a pool of 3 plants where the four first leaves were infiltrated with 0.5 mL infiltration buffer using a 1 mL needleless syringe. For the RNA-Seq experiment, plants were divided into two groups, p103::MD05G1011100/pBIN61-p19 (overexpression) and p103-empty/pBIN61-P19 (control) with 4 biological replicates each and were collected 4 days after infiltration. For the time-course targeted qPCR experiment, plants were divided into two groups, p103::MD05G1011100/pBIN61-p19 and p103-empty/pBIN61-P19 with 3 biological replicates each and were collected 0, 1, 2, 3, 4 and 7 days after leaf infiltration. Finally, for the analytical chemistry analysis, 3 groups of 4 biological replicates were used: at day 0, a first group was collected as not infiltrated T=0 control and two additional groups of infiltrated leaves were collected after 5 days (p103::MD05G1011100/pBIN61-p19 and p103-empty/pBIN61-P19). All samples were flash frozen in liquid nitrogen directly after collection.

### Library preparation and sequencing

cDNA libraries were prepared from 20 ng mRNA using the SMARTer Stranded RNA-Seq kit according to the manufacturer’s guidelines (ClonTech laboratories, Mountain View, CA, USA). Libraries were analyzed and quantified as described in Legay et al. (2016). The pooled libraries were sequenced on an Illumina MiSeq using 4 consecutive runs (Illumina MiSeq reagent V3-150 cycles) to generate 76 base-pairs paired-end reads. FASTQ files were imported in pairs in CLC genomics workbench v8.0.1 discarding poor-quality reads (<Q30). For each library, reads were trimmed and filtered using the following criteria: sequence quality <0.01, no ambiguous nucleotides, minimum read length >35 nucleotides, trimming against the Illumina adaptor sequence, and finally a hard trim of 10 nucleotides at the 5’ end and 2 nucleotides at the 3’ end. The filtered reads were mapped to the *N. benthamiana* transcriptome v5-primary transcript (Nakasugi et al., 2014) obtained from the *N. benthamiana* genome and transcriptome website (http://benthgenome.qut.edu.au/) with the following criteria: a mismatch, gap and insertion cost on the maximum settings (3=stringent mapping), reads should have 80% identity and 90% coverage to the reference transcriptome. An additional annotation of the transcriptome was performed against the *A. thaliana* database using BLAST2GO Pro v3.0. Expression values were calculated using the RPKM (Reads per kilobase transcript per million reads) method (Mortazavi et al., 2008). In order to determine the differentially expressed genes between the tobacco leaves infiltrated with p103::MD05G1011100/pBIN61-p19 and p103-empty/pBIN61-P19, a Baggerley’s ‘on proportions’ weighted test (Baggerly et al., 2003) combined with a false discovery rate correction (Benjamini-Hotchberg) set at 0.05 was used. Expression cut-off values were set at 2-fold increase/decrease and 10 RPKM difference, respectively. A gene ontology enrichment analysis was performed from the significantly regulated genes in Cytoscape (v3.6.0) with the ClueGO v2.5 and CluePedia v1.5 plugins (Bindea et al., 2009) to highlight regulated biological processes with the following criteria: gene ontology from level 5 to 7, kappa score set at 0.4, only biological process with at least 8 genes or 4% of the genes were retained, FDR corrected significance (Benjamini-Hotchberg) of the biological process has been set at 0.05. Raw sequences have been deposited at the NCBI Gene Expression Omnibus website (GEO, http://www.ncbi.nlm.nih.gov/geo, accession number: GSE212828).

### Total lignin quantification and lignin monomer composition

The following procedure has been already described in previous work (Yamamura et al., 2010; Behr et al., 2018). Total lignin content has been assessed on cell wall residue preparations (CWR) obtained from 4 biological replicates from (i) non infiltrated leaves sampled at T=0 day after infiltration, (ii) leaves infiltrated with a mixture of *A. tumefaciens* strains transformed with p103::MD05G1011100 and pBIN61-p19 (Overexpression) at T=5 days after infiltration and finally (iii) leaves infiltrated with a mixture of *A. tumefaciens* strains transformed with an empty p103 vector and pBIN61-p19 (Control) at T=5 days after infiltration. Briefly, CWR were obtained by washing the powdered plant material first with methanol (80% v/v) under agitation for 4 h, followed by five additional vortexing/centrifugation cycles with ethanol (80% v/v). After drying, 5 mg of CWR were digested with 2.6 mL of 25% (v/v) acetyl bromide in glacial acetic acid for 2 h at 50°C using a Hach LT200 system. After digestion, the solution was transferred to a 50 mL Falcon tube containing 10 mL of 2 M sodium hydroxide and 12 mL of glacial acetic acid. The reaction tube was rinsed with glacial acetic acid and 1.75 mL of 0.5 M hydroxylammonium chloride was added. Finally, the total volume was adjusted to 30 mL with glacial acetic acid. The absorbance of the solution was read at 280 nm in a spectrophotometer, with an extinction coefficient of 22.9 L·g^−1^·cm^−1^ for lignin determination.

For the characterization of lignin monomers, 10 mg of dried CWR were placed in a 10 mL borosilicate glass tube with 2 mL of NaOH 2M and 30 µL of nitrobenzene. The tubes were closed and heated to 165°C for 1h (Hach LT200 system). Samples were left cooling at ambient temperature then on ice for several minutes. Ten µL of vanillin-D3 at 10 mg/mL were added as recovery standard and, after centrifugation, 1500 µL of supernatant were collected in 5 mL microcentrifuge tube. Nitrobenzene was removed by four successive washings with 1 mL of ethyl acetate. The pH of the remaining aqueous phase was lowered to 2-3 by adding 240 µL of HCl 6M. The oxidation products were recovered by three successive extractions with 1 mL of ethyl acetate. The combined organic layers were washed with 500 µL of saturated aqueous NaCl solution then dried over anhydrous Na_2_SO_4_.

Prior to GC-MS analyses, the oxidation products were derivatized by trimethylsilylation using the following protocol: ten µL of salicylic acid-D4 at 10 µg/mL were added as internal standard in a 2 mL glass vial with 50 µL of the extract and the mixture was dried under a gentle nitrogen stream. 50 µL of BSTFA : TMCS mixture (Bis(trimethylsilyl)trifluoroacetamide, Trimethylchlorosilane, 99:1 v/v) were added to the extract and the reaction was carried on at 60°C for 30 min.

Quantitative analyses were performed by GC-MS using a HP-5MS column (30m x 0.25mm, 0.25µm, Agilent) installed in a 7890B-5977A instrument (Agilent). A volume of 1 µL was injected at 250°C in splitless mode. The oven program started at 40°C for 5 minutes, increased to 230°C at 10°C/min, then to 320°C at 40°C/min and was kept at 320°C for 10 min. Quantitative results were provided in Selected Ion Monitoring mode thanks to internal calibrations

### Sample preparation for optical microscopy

Leaves (sections of 1 cm of diameter) were cut from tobacco plants agroinfiltrated with the binary construct containing the p103::MD05G1011100 and pBIN61-p19and controls (infiltrated with P103 empty vector/pBIN61-p19). The samples were plunged in 2 ml of fixation solution (50 ml of NaH_2_PO_4_/Na_2_HPO_4_ 0.2 M at pH 7.2, 20 ml of paraformaldehyde 10% (v/v), 2 mL of glutaraldehyde 1% (w/v), 1 g of caffeine and 28 mL H2O). The sections were subjected to vacuum infiltration for 10 min at 600 mbar and then the fixation solution was removed and replaced with 2 mL of EtOH 70% (v/v) and stored at 4°C until further processing. The tissues were then dehydrated with EtOH (95% v/v for 30 min, 95% v/v for 1 h, 100% v/v for 30 min and 100% v/v for 2 h). The tissues were thereafter transferred for 2 h into a solution containing in a 1:1 ratio EtOH 100% v/v and a soaking solution composed of resin 100 mL (Technovit 7100, Kulzer Technik, Wehrheim, Germany), 1 g initiator, 2 mL of ethylene glycol and 10 mL of PEG 400. Finally, the samples were left for 24 h in 100% of the soaking solution. One mL of hardening solution (Technovit 7100, Kulzer Technik, Wehrheim, Germany) was then added to the soaking solution and dispensed into histomolds (Leica Biosystems, Nussloch, Germany) where the samples were included. Moulds were then placed in an oven at 37°C until complete hardening of the resin.

Cross-sections of 10 μm thickness were cut with a microtome (Leica Biosystems, Nussloch, Germany), put on slides and incubated for 15 min in FASGA solution (3 mL Safranine 1% (w/v), 14 mL Alcian Blue 8 GX, 1 mL acetic acid, 30 mL glycerine and 20 mL H_2_O) at room temperature, then rinsed 3 times for 5 min with H_2_O and observed.

Sections were also incubated with the LM10 probe (Behr et al., 2016). Briefly, the LM10 antibody was diluted 10-fold in milk protein (MP)/PBS 5% (w/v). Sections were thereafter incubated at room temperature with the LM10 antibody for 1.5 h, rinsed three times for 5 min in PBS and incubated at room temperature for 1.5 h with the anti-rat IgG coupled to FITC (Sigma) diluted 100-fold in MP/PBS. Prior to observation, three washings with PBS were performed for 5 min.

Images were collected using an optical microscope (Olympus BX51, Tokyo, Japan) equipped with a GFP filter, with a Toupview camera and software (ToupTek Photonics, Zhejiang, China). The images were imported into ImageJ 1.53K software (http://imagej.nih.gov.ij). Image processing was carried out as follows (Hartig, 2013). As a first step, the image pixels were converted to µm using the command “Set Scale” to measure the area. Then the area, integrated intensity and mean grey value were ticked from the command “Set Measurements”. Fluorescence intensity and area were quantified from 15 elements of 2 images using the “Freehand selections” command. The mean total fluorescence of the 15 elements selected from the 2 images was expressed as integrated intensity (IntDen). In order to correct for background fluorescence, the corrected total cell fluorescence (CTCF) was also shown and expressed in arbitrary units (a.u.). It was calculated as follows: IntDen – (area of selected element x mean fluorescence of background readings). Furthermore, to compare the distribution of the fluorescent signal (converted to grey values) among the images, the function “Histogram” was used. The x-axis represents the occurrence of grey values and the y-axis shows the number of pixels found for each grey value. The histograms were also rendered in log-scaled values.

## Results

### Phylogenetic analysis and functional annotation of the MD05G1011100 – MYB transcription factor

**Figure 1:**
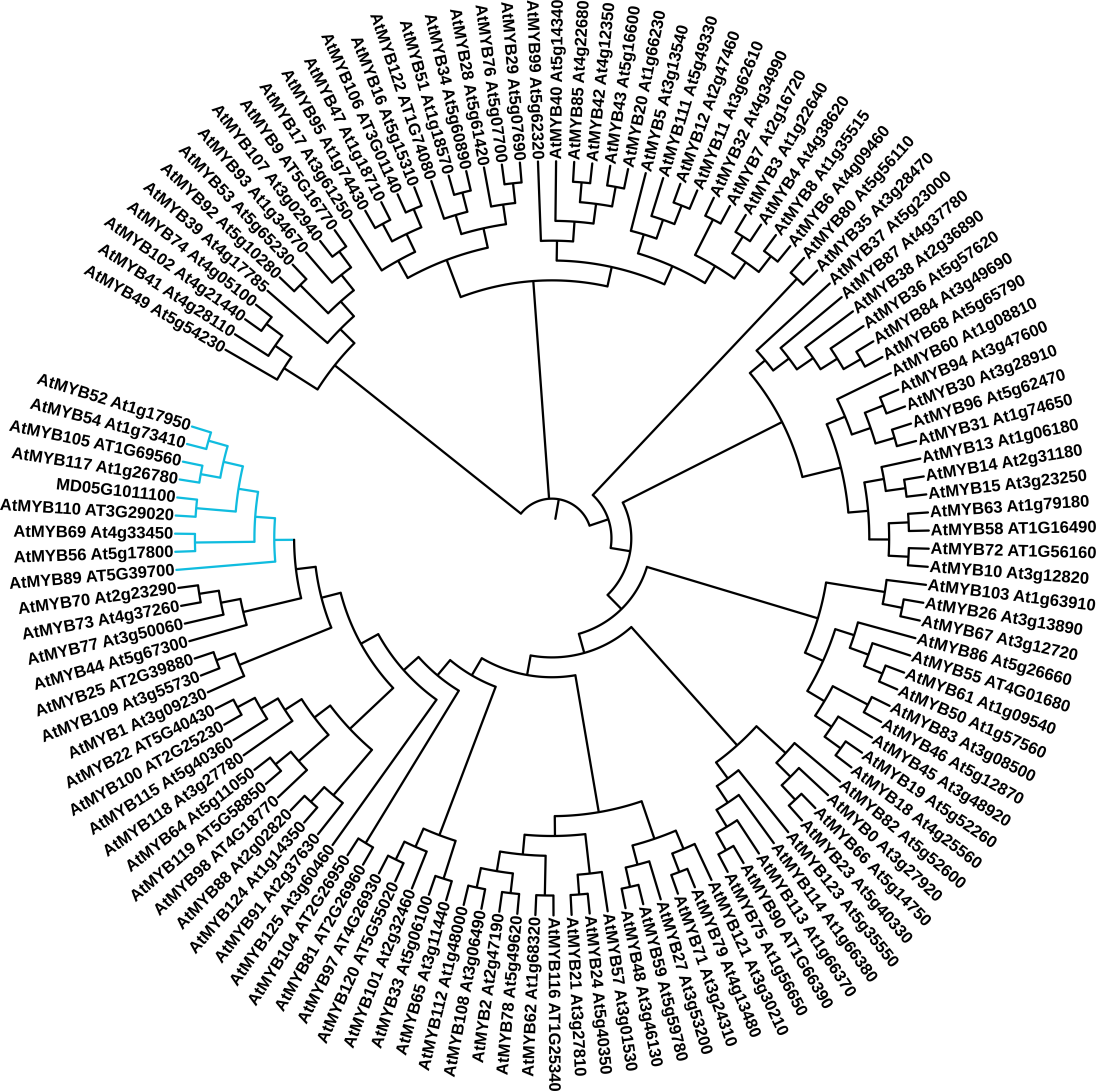
Phylogenetic analysis of the apple MD05G1011100 MYB transcription factor. The predicted amino acid sequences of these gene were compared to the 126 MYB transcription factor sequences identified in *Arabidopsis thaliana* using the MUSCLE algorithm. The cladogram was generated using neighbor-joining algorithm from in ITOL (itol.embl.de). The clade containing the MD05G1011100, AtMYB52, AtMYB54, AtMYB105, AtMYB117, AtMYB110, AtMYB69, AtMYB56 and AtMYB89 is highlighted in blue.

The MD05G1011100 gene was first identified in our bulk gene expression profiling study, which compared the expression of genes in both russeted and waxy apple fruit skin (Legay et al., 2015). This work was performed using the first draft of the apple genome (Velasco et al., 2010) where MD05G1011100 was first identified as MDP0000852158 (100% identity), a Myb domain protein 52 transcription factor (*Arabidopsis thaliana*) gene located on the chromosome 5. This contig also shares high identity (90.20%) and coverage (100%) of nucleotide sequence with MDP0000291518, which could be due to the redundancy in the apple genome (Velasco et al., 2010). To better define the MD05G101110 functional annotation, we compared its protein sequence against Arabidopsis MYB transcription factor sequences using a phylogenetic approach. Three different algorithms were used namely: MUSCLE ( (Edgar, 2004), **Figure 1**), Clustal Omega (Sievers and Higgins, 2014) and COBALT (Papadopoulos and Agarwala, 2007) (**Supplementary Figure 1**) alignment tools combined with a neighbor joining clustering method. These three approaches showed slightly different results. We identified a group constituted by 8 transcription factors, in which MD05G1011100 is clustered. These are AtMYB52, AtMYB54, AtMYB105, AtMYB117, AtMYB110, AtMYB69, AtMYB56 and AtMYB89. Using the MUSCLE algorithm, MD05G1011100 was clustered with AtMYB110, whereas it is clustered with MYB105/MYB117 and MYB69 using the Clustal Omega and COBALT algorithms, respectively. The GDR Rosaceae portal (Jung et al., 2019) also provides homologies of MD05G1011100 against multiple databases: uncharacterized protein (M5VN75_PRUPE, *Prunus persica* 77.05% identity, v.s. ExPASy TrEMBL), Predicted: transcription factor MYB44-like (100% identity, *Malus domestica*, NCBI nr database), MYB52 (AT1G17950, *A. thaliana*, 83.64% identity, v.s. ExPASy Swiss-Prot and TAIR10). Result obtained from the MD05G1011100 amino acid sequence using the NCBI BlastX also showed 83.64% identity with AtMYB52 (AT1G18850) with only 31% coverage, in the first 120 amino acids, which correspond to the MYB-binding domain and appears conserved between *M. x domestica* and *A. thaliana*. The remaining sequence seems to be not conserved. A deeper analysis of the amino acid sequence using the Phyre2 (Protein Homology/analogY Recognition Engine V 2.0, **Supplementary Figure 2**) revealed that this non-conserved region corresponds to an intrinsically disordered region (IDR), for which the function is not clearly defined but might be involved in protein-protein interaction (Babu, 2016). Interestingly, such observation has already been made in our previous work on MdMYB93, which reinforce a putative functional role of IDRs in plant MYB domain transcription factors. For better readability, MD05G1011100 will be named MdMYB52 transcription factor in the following sections.

### Specific expression of MdMYB52 transcription factor in russeted apple fruit skin

**Figure 2:**
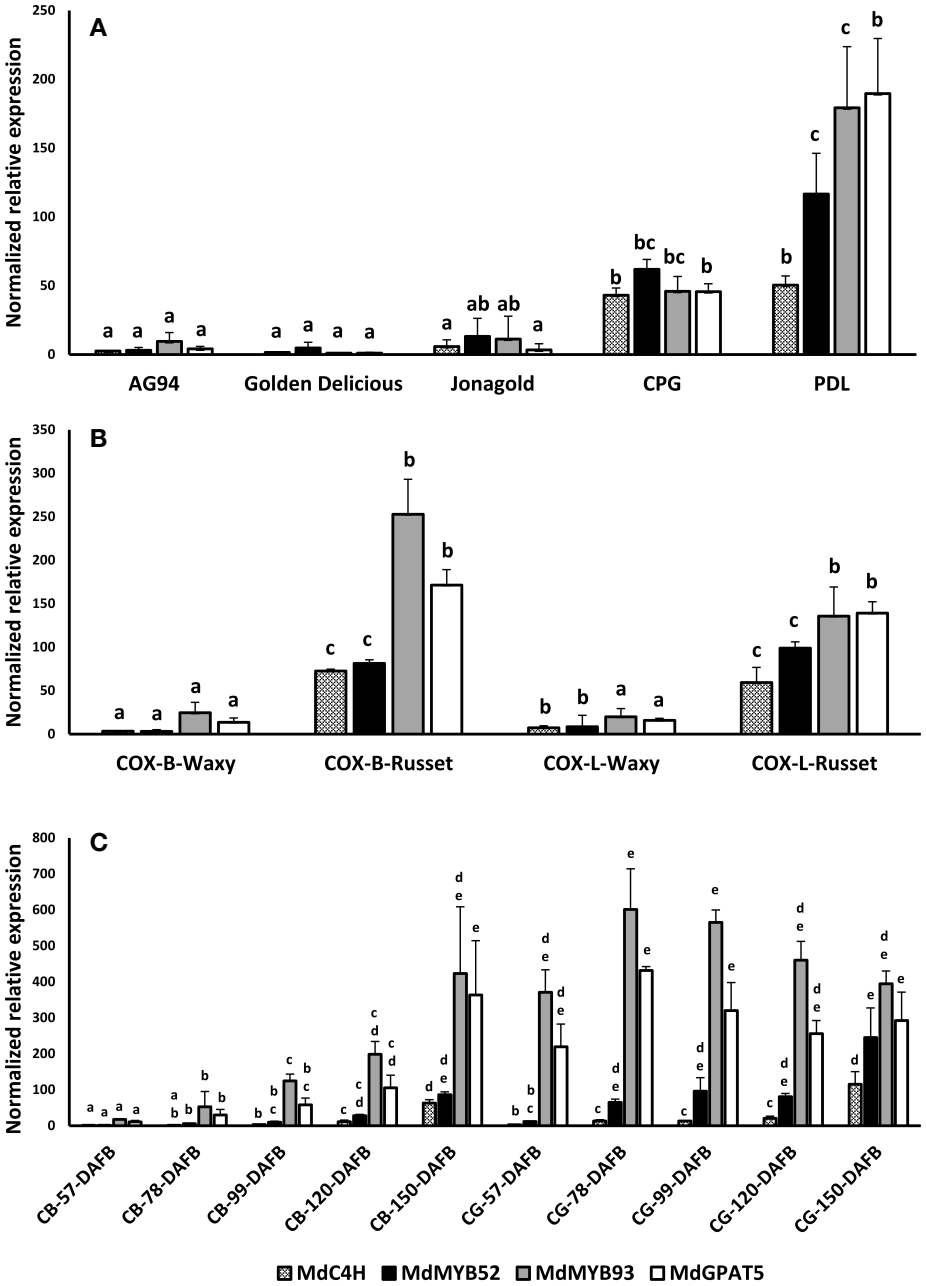
Targeted gene expression profiling of MdC4H, MdMYB52, MdMYB93 and MdGPAT5 in apple fruit skin tissues. **(A)** profiling in waxy (AG94, Golden delicious, Jonagold) and russeted (Court-Pendu-Gris, CPG and Patte de Loup, PDL). **(B)** profiling in Cox Orange Pippin (COX) waxy and russeted tissues collected from Belgium (B) and Luxembourg (L) locations. **(C)** Profiling in two semi-russeted and russeted nearly isogenic varieties, Reinette du Canada Blanche (CB) and Reinette du Canada Grise (CG), respectively. DAFB: Days after full bloom. Statistical analysis was performed independently for each dataset and gene from log2 transformed data using an ANOVA one-way followed by a Tukey’s *Post-Hoc* test. Letters display the significantly different groups.

To assess the involvement of MdMYB52 in russeting and, by extension, in the suberization process, a gene expression profiling was performed on different apple genotypes and apple skin tissues. Apart from MdMYB52, we also included genes previously identified to be involved in the suberization process in apple fruit skin (Legay et al., 2015; André et al., 2022). This includes MdC4H, a core gene of the phenylpropanoid pathway, MdMYB93, a suberin biosynthesis master regulator (Legay et al., 2016) and MdGPAT5, a core gene being involved in the biosynthesis of the aliphatic domain of suberin (Legay et al., 2015). We first quantified the MdMYB52 TF expression in waxy (CRAW-AG94, Golden Delicious and Jonagold) and russeted (Court-pendu grise, Patte de Loup) varieties and observed that the expression of MdMYB52 TF was significantly higher in the russeted apple varieties (**Figure 2A**). This specific expression seems to be tightly linked to suberized tissues as we also observed an increased expression of MdMYB52 in the russeted skin areas of the semi-russeted (patchy) ‘Cox Orange Pippin’ variety compared the waxy skin areas (**Figure 2B**). Finally, we investigated the MYB52 expression pattern during the fruit growth of ‘Reinette du Canada Blanc’ (CB) and ‘Reinette Grise du Canada’ (CG) (André et al., 2022). CB is a slightly russeted variety, which displays an increasing russet phenotype during fruit growth; CG is a heavily russeted apple. It is noteworthy that these two varieties can be considered as nearly isogenic varieties as the SSR marker fingerprinting did not show any differences (André et al., 2022). From these samples the expression of MdMYB52 was only partially correlated with the expression of the key suberin biosynthesis and regulatory genes, MdGPAT5 and MdMYB93, respectively (**Figure 2C**). As an example, in CB, we observed that MdMYB52 and MdMYB93 were highly correlated in expression (R^2^ = 0.92, p-value< 0.0001, Pearson correlation) whereas they were not in CG (R^2^ = -0.12, p-value=0.6604, Pearson correlation). Despite MdMYB52 expression was clearly linked to the suberization of the apple fruit skin, its expression reached its maximum at the commercial harvest time-point (150 days after full bloom). Finally, looking at the overall gene expression profiles, MdMYB52 showed a strong correlation with MdC4H (R^2^ = 0.89, p-value<0.0001) and a significant, but lower correlation, with MdMYB93 (R^2^ = 0.61, p-value<0.0001) and MdGPAT5 (R^2 =^ 0.52, p-value<0.0001) (**Supplementary Table 1**)

### Whole gene expression profiling of *N. benthamiana* leaves overexpressing the MdMYB52 transcription factor

To characterize its function, MdMYB52 transcription factor was transiently expressed in a heterologous system, namely *N. benthamiana*, and compared to control leaves infiltrated with an empty vector. Five days after infiltration, leaves expressing the MdMYB52 transcription were similar to the control leaves (**Supplementary Figure 3**). However, 8 days after infiltration, a strong difference in phenotype was observed. Leaves expressing MdMYB52 displayed some transparent areas with a “loss of green”, which reminded stress induced-hypersensitive response phenotypes (**Supplementary Figure 3**).

To better understand the causes linked to this strong response, eight libraries were prepared from RNA samples and sequenced using a NextSeq500 Illumina sequencer. Libraries were successfully mapped against the *N. benthamiana* Nbv5 primary transcriptome with a rate ranging from 72 to 75% (**Supplementary Table 2**). After a statistical analysis, fold change and minimum expression cut-off comparing Nicotiana leaves overexpressing MdMYB52-like to those infiltrated with empty vector, 2158 differentially expressed genes were retained with an equal repartition of positively and negatively regulated genes, 52.1% and 47.9% respectively (**Supplementary Table 3**).

**Figure 3:**
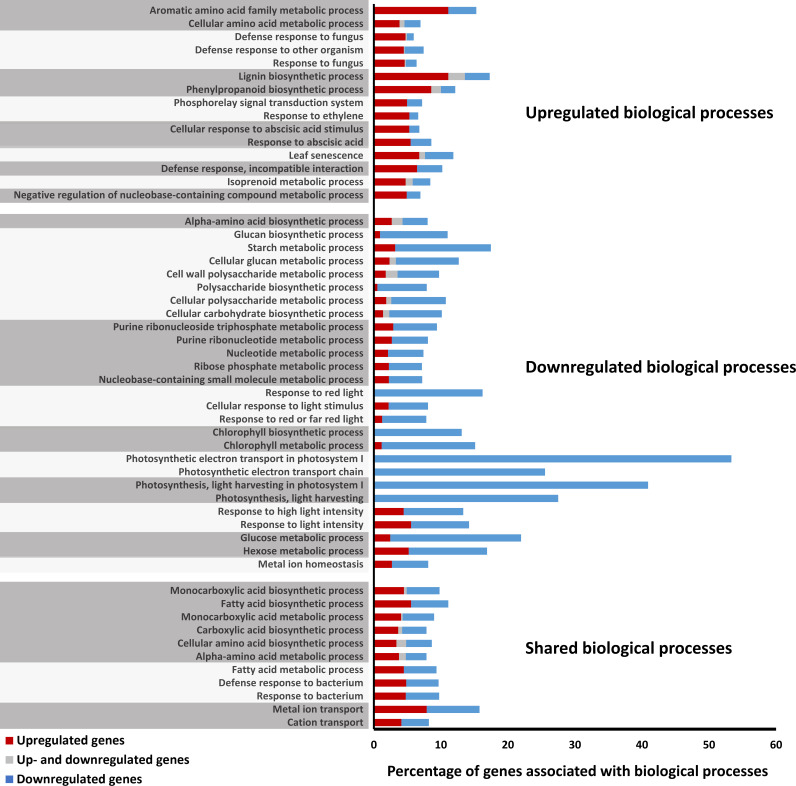
Gene ontology enrichment analysis of the significantly regulated genes obtained after transient overexpression of the MdMYB52 transcription factor in *Nicotiana benthamiana* leaves after 5 days (-2>log_2_ ratio (MdMYB52 TF/Empty vector)>2, FDR corrected p-value<0.05). Orthologous TAIR codes were used in Cytoscape (v3.1.0) with the ClueGO v2.1.1 and CluePedia v1.1.1 plugins to highlight the significant biological processes (Benjamini-Hotchberg corrected p-value<0.05, gene ontology from level 5 to level 7, GO terms grouping option activated, kappa score set at 0.4). For each significant biological process, the percentage of implicated genes is displayed. Percentage of upregulated, downregulated genes as well as genes which have been found up and downregulated are displayed in red, blue and grey, respectively. GO terms grouping is displayed using alternate light and dark grey color over GO terms. Detailed results are available in **Supplementary Table 4**.

To further elucidate the function of MdMYB52, a gene ontology analysis was performed to highlight differentially regulated biological processes (**Figure 3**, **Supplementary Table 4**). Firstly, a massive downregulation of genes linked to the photosynthesis, electron transport and chlorophyl biosynthesis was observed. This included *Photosystem I reaction center subunit V*, *Oxygen-evolving enhancer protein 2-2*, *Chlorophyll a-b binding protein 6A* or *Ribulose bisphosphate carboxylase/oxygenase activase* genes to name a few and further suggested that an important stress occurred in leaves expressing MDMYB52 (Bilgin et al., 2010; Evers et al., 2010). This trend was also confirmed by a concomitant decrease in expression of genes linked to the glucose and hexose metabolic process and the ribonucleotide metabolic process, suggesting a clear slowdown in the primary metabolism. Finally, and concomitantly, a large number of biological processes gathered into a large polysaccharide metabolism group was also downregulated. This group includes GO-terms such as glucan and starch biosynthesis or cell wall polysaccharide metabolic processes, which seemed to be directly impacted by the decrease of photosynthesis observed previously. More particularly, the primary cell wall metabolism was also particularly impacted upon transient expression of MdMYB52 transcription factor. As an example, several genes coding for some xyloglucan endotransglucosylase/hydrolase (XTH), cellulose synthase-like, fasciclin-like arabinogalactan protein 1 and 17 were strongly down-regulated. Altogether, the down-regulated biological processes observed here could be associated with global stress responses observed during abiotic stresses in plants (Evers et al., 2010; Nouri et al., 2015).

Up-regulated biological processes also confirmed this trend. A large number of stress responsive genes were found including heat shock proteins, glutathione-*S*-transferase tau, osmotins, pathogenesis related proteins, dehydrins, and late embryogenesis abundant proteins (LEA). Such genes are induced upon multiple abiotic stresses including drought, salt or cold stresses. Glutathione-*S*-transferase (GSTs) expression is triggered in response to multiple stresses. It is well documented that these are participating in the regulation of oxidative stress (Kumar and Trivedi, 2018). Osmotins are known to be important actors of abiotic stress tolerance in plants such as drought, cold, salinity stress by helping the accumulation of proline, which is quenching reactive oxygen species and free radicals (Anil Kumar et al., 2015). At the regulatory level, stress-related signal transduction pathways seemed to be altered with an increased expression of multiple calmodulin (CML37, CML38, CML41, CML44, CML45, CML49, PBP1, …), calcium dependent protein kinase (CPK) and protein phosphatase 2C (25, 37 and 40) genes. This was correlated by a concomitant induction of genes coding for heat shock transcription factors or dehydration-responsive element-binding (DREB), which are considered as key regulators of abiotic stress tolerance in plants (Agarwal et al., 2006). Altogether, our transcriptomic data showed the increased expression of several genes involved in biological processes associated with plant stress responses, in leaves expressing MdMYB52. More particularly these stress responses remind those observed during plant responses to osmotic or oxidative stresses.

Furthermore, the gene ontology analysis also highlighted biological processes associated with leaf senescence, which could explain the strong phenotype observed in leaves expressing MdMYB52 TF. Notably, we found several WRKY transcription factors, i.e., WRKY6, WRKY22, WRKY42, WRKY53 (FC=4.11, 25.46, 2.11 and 13.59, respectively), which have been previously described to be crucial actors of the leaf senescence and programmed cell death in plants (Robatzek and Somssich, 2002; Miao et al., 2004; Zhou et al., 2011; Zentgraf and Doll, 2019; Niu et al., 2020). As an example, WRKY53 acts as one hub in the complex senescence regulatory network and its expression is tightly regulated by either H_2_O_2_ and hormones such as jasmonic acid or salicylic acid (Zentgraf and Doll, 2019). FMO1 gene, which is involved in the regulation of immunity and cell death in Arabidopsis, was also upregulated in leaves expressing the MdMYB52 transcription factor (Bartsch et al., 2006). A recent study showed that FMO1 is involved in the regulation of systemic cell death during excess of light, systemic acquired resistance and systemic acquired acclimation mechanisms (Czarnocka et al., 2020). Authors showed that FMO1 specifically regulates the spread of reactive oxygen species systemic signal and is required for the induction of ascorbate peroxidase (APX2) and ZAT10 during stress treatments. Interestingly, we also found upregulated genes similar to ZAT10 and ZAT11 in leaves expressing MdMYB52. In Arabidopsis, previous studies showed that ZAT10 acts as both negative and positive regulator of plant defenses against abiotic stresses and more particularly as a mediator of reactive oxygen species production (Mittler et al., 2006). Similarly, ZAT11 is involved in the regulation of the oxidative stress-induced programmed cell death (PCD) (Qureshi et al., 2013).

Summarizing all previous observations, MdMYB52 expression in Nicotiana leaves triggers a massive oxidative stress response associated with senescence or programmed cell death. We further investigated our dataset to try to identify what could cause this stress response.

Interestingly, in our gene ontology analysis, we found significantly upregulated biological processes associated with lignin, phenylpropanoid and aromatic amino acid biosynthesis. Indeed, a large number of peroxidase (PRX) genes including lignin-forming peroxidase, PRX4, PRX51, PRX52, PRX72 genes were strongly upregulated in leaves expressing MdMYB52. It is noteworthy that a gene annotated as a suberization-associated anionic peroxidase (SAAP), similar to PRX52, was also induced. In potato, this SAAP showed a preference for feruloyl (*o*-methoxyphenol)-substitutes substrates (Bernards et al., 1999). Some models suggest that the polyphenolic domain of suberin can be considered as a lignin-like polymer enriched with feruloyl moieties (Bernards, 2002). Remarkably, other key actors of the lignin biosynthesis pathway were also induced during the transient overexpression of MdMYB52 TF. This includes mostly a number of genes belonging to the phenylpropanoid biosynthesis pathway: 4-coumarate-CoA ligase (4CL), cinnamate-4-hydroxylase (C4H), caffeic acid *O*-methyltransferase (OMT1), hydroxycinnamoyl-coenzyme A shikimate/quinate hydroxycinnamoyltransferase (HCT), CCoAMT1, cinnamyl-alcohol dehydrogenase (CAD) genes, and suggests an enhanced biosynthesis of lignin precursors (G- and S-units) which might be further used by peroxidases to build the lignin polymer (Fraser and Chapple, 2011). Several tyramine *N*-feruloyltransferase, responsible for the biosynthesis of *N*-feruloyltyramine, were induced in Nicotiana leaves overexpressing MdMYB52 (FC=6.86). Feruloyltyramine has been previously described as a major component of the polyphenolic domain of suberin (Bernards et al., 1995; Bernards et al., 1999). More recently, in tomato, feruloyltyramine has been described as a crucial component of ligno-suberin deposition in vascular cell walls decreasing pathogen colonization (Kashyap et al., 2022). In addition to this, we observed several Casparian-like protein genes (CASP-like protein PIMP1, CASP5, CASPIN26). Higher expression of CASP-like proteins was also observed in russeted apple skin, as well as in *N. benthamiana* during transient expression of MdMYB93, a suberin biosynthesis master regulator (Legay et al., 2015; Legay et al., 2016). These are suspected to be involved in the structural organization of the suberin aliphatic domain, as well as its linkage to the lignin-like/polyphenolic domain during the suberization process, but their exact function is still under investigation (Woolfson et al., 2022). An upregulated laccase 14 gene was also found in our dataset (FC= 14.48). Laccases are thought to be involved in many processes including maintenance of cell wall structure, defense against biotic and abiotic stresses, wound healing, iron metabolism and polymerization of phenolic compounds (Janusz et al., 2020). More particularly, in poplar, LAC14 has been described as a key player of the guaiacyl lignin deposition, which has been also described as an important component of the polyphenolic domain of suberin (Bernards et al., 1999; Graça, 2015; Qin et al., 2020; Woolfson et al., 2022). Concomitantly, a large number of upregulated genes coding for xyloglucan endotransglucosylase/hydrolase (XTH) proteins, expansins (EXP) and pectin methyl esterase inhibitor (PMEI) were obtained. These genes were also observed in Nicotiana leaves expressing the suberin master regulator MdMYB93, as well as in russeted (suberized) apple fruit skin (Legay et al., 2015; Legay et al., 2016). Higher expression of XTH and EXP might be associated with cell wall loosening (Gall et al., 2015). In the apple fruit model, we hypothesized that, during the suberization process, this massive expression of hemicellulose modification genes, including XTH, PMI, EXP, associated with the above-mentioned lignification process could be considered similar to secondary cell wall biogenesis, which facilitates the anchoring of the aliphatic domain of suberin onto the cell wall (Legay et al., 2015). However, this scheme is not fully clear, we also observed an increased expression of Xylem NAC Domain 1 (XND1) in leaves expressing MdMYB52. XND1 is a crucial negative regulator of secondary cell wall biogenesis and programmed cell death in Arabidopsis (Zhao et al., 2008). This observation is opposed to the statement described above, but one explanation could be that the XND1 expression somehow mediates the massive increase of lignin biosynthesis and cell wall modification genes, but it is difficult to provide any robust hypothesis from a global transcriptomic point of view.

In summary, whole transcriptomic data showed that the transient overexpression of MdMYB52 in Nicotiana leaves triggered a massive stress response associated with leaf senescence and programmed cell death components. MdMYB52 seemed to play a central role in the regulation of phenylpropanoid and lignin biosynthesis. This strong trend was also confirmed by the significant induction of biological processes linked to the metabolism of aromatic amino acids, precursors of the phenylpropanoid pathway. It is still difficult to link these observations. MdMYB52 overexpression might enhance the activity of lignin biosynthesis actors such as PRXs, the latter generating an uncontrolled increase in ROS production leading to this stress response, or another hypothesis could be that MdMYB52 regulates both lignin biosynthesis and programmed cell death in suberized cells. The present RNA-Seq data provides a snapshot of the MdMYB52 regulatory functions 5 days after infiltration, it is, thus, difficult to clearly distinguish the chronology of these different events. To better answer this question, we performed a targeted time-course gene expression analysis on a subset of genes belonging to each of the biological processes described above.

### Targeted time-course gene expression analysis of some MdMYB52 regulated genes

**Figure 4:**
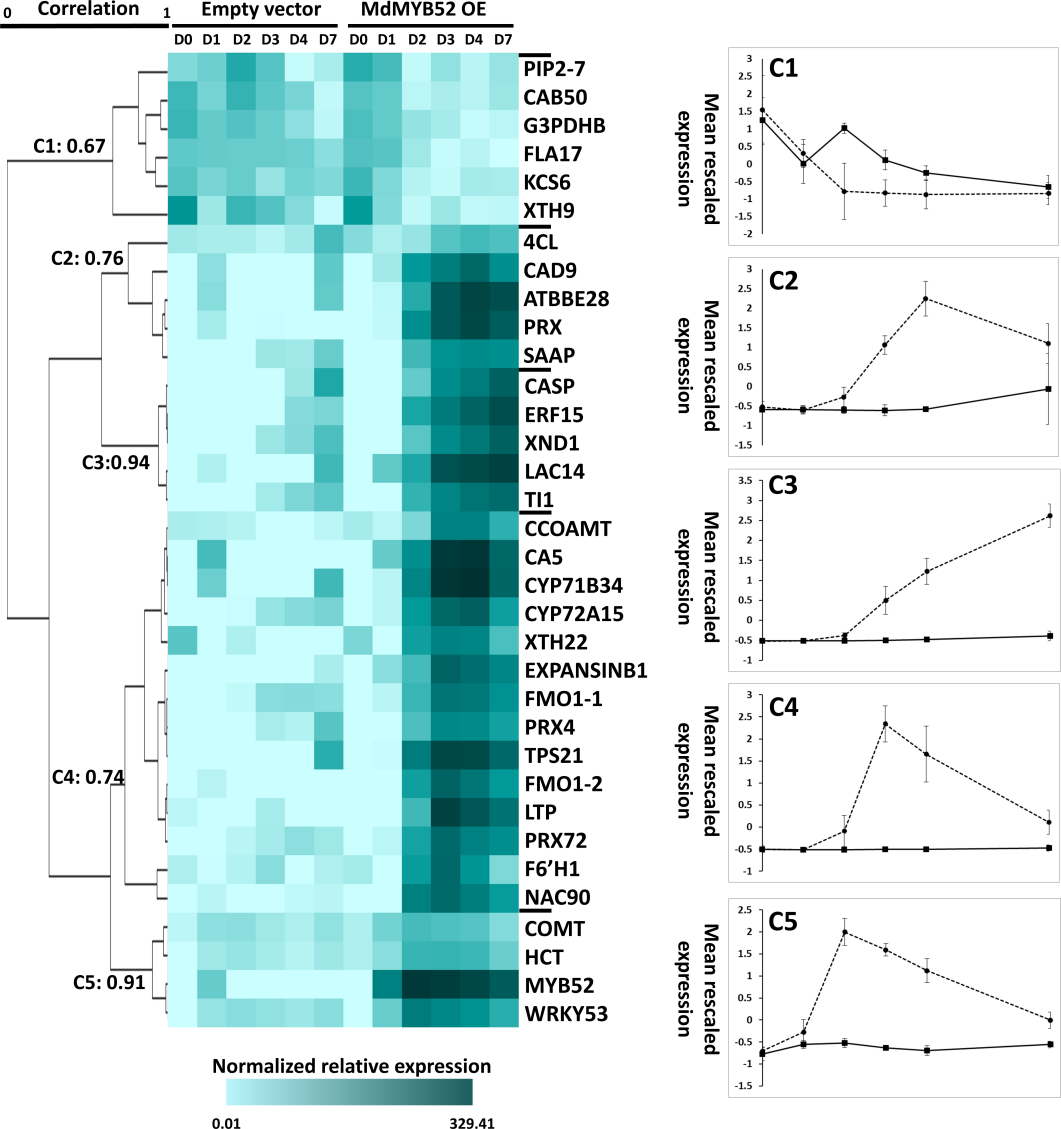
Time-course targeted gene expression analysis performed in *Nicotiana benthamiana* leaves expressing the MdMYB52 transcription factor compared to the empty vector (control), 0, 1, 2, 3, 4 and 7 days after infiltration. Genes were selected from the significantly regulated genes observed in the whole gene expression profiling study. A hierarchical clustering was performed using uncentered Pearson correlation and a complete linkage agglomeration method. Clusters were defined using a minimum Pearson correlation of 0.65. TPS21: alpha-humulene/(-)-(E)-beta-caryophyllene synthase, LTP: Lipid Transfer Protein, FMO1-1: Flavin-containing monooxygenase 1 (copy 1), FMO1-2 Flavin-containing monooxygenase 1 (copy 2), CYP72A15: Cytochrome P450 family 72, subfamily A15, XND1: xylem NAC domain 1, WRKY53: WRKY Transcription Factor 53, F6’H1: Fe(II)- and 2-oxoglutarate-dependent dioxygenase family, PRX72: Peroxidase 72, CAD9: cinnamyl alcohol dehydrogenase 9, PRX: Peroxidase superfamily protein, CYP71B34: Cytochrome P450 family 71, subfamily B34, NAC90: NAC transcription factor 90, CA5: Carbonic Anhydrase, LAC14: Laccase 14, ERF15: Ethylene response Factor 15, TI1: Trypsin inhibitor 1-like, ATBBE28: FAD-binding Berberine family protein, ExpansinB1: Expansin-like B1, CASP: CASP-like protein IN26, XTH22: Xyloglucan endotransglucosylase/hydrolase protein 22, CCOAOMT: Probable caffeoyl-CoA *O*-methyltransferase, PRX4: Peroxidase 4, SAAP: Suberization-associated anionic peroxidase, COMT: Caffeic acid 3-*O*-methyltransferase, 4CL: 4-coumarate–CoA ligase-like 10, HCT: Hydroxycinnamoyl-Coenzyme A shikimate/quinate hydroxycinnamoyltransferase, KCS6: 3-ketoacyl-CoA synthase 6: FLA17: Fasciclin-like arabinogalactan protein 17, PIP2-7: Aquaporin PIP2-7, CAB50:Chlorophyll a-b binding protein 50, chloroplastic, XTH9:Xyloglucan endotransglucosylase/hydrolase protein 9, G3PDHB: Glyceraldehyde-3-phosphate dehydrogenase B, chloroplastic.

The expression of a total of 34 genes were tested using qPCR at 0, 1, 2, 3, 4 and 7 days after infiltration by *A. tumefaciens*/P103::MdMYB562 and *A. tumefaciens*/empty 103 vector (**Figure 4**, **Supplementary Table 1**). A hierarchical clustering using Pearson uncentered correlation distance and complete linkage method, was performed from the normalized relative expression measured for each gene. Using a 65% correlation cut-off value, 5 clusters were defined. One cluster displays genes which were downregulated during MdMYB52 transient expression, the four other clusters display genes that were instead more expressed during MdMYB52 overexpression; it is noteworthy that these clusters were defined according to their maximum expression within the genes. Cluster 5 displays genes which are reaching their maximum expression 2 days only after infiltration and it includes MdMYB52 which showed, as expected, a massive expression at day 2 (FC= 5866.27, p-value<0.0001). Three other genes also belong to this cluster, COMT, HCT and WRKY53, suggesting that MdMYB52 is involved in the regulation of the phenylpropanoid pathway and senescence/PCD. Cluster 4 and cluster 2 showed a delayed but similar trend, with a maximum expression observed 3 and 4 days after infiltration, respectively. In these clusters, we observed multiple key members of the phenylpropanoid and lignin biosynthesis pathway. These include CCoAMT, 4CL, CAD9, as well all the peroxidases PRX, PRX4, PRX72, the suberin associated anionic peroxidase (SAAP) and confirmed the important role of MdMYB52 in the regulation of the lignin deposition during the suberization process. As expected, the expression of expansins, xyloglucan endotransglucosylases/hydrolases and lignin biosynthesis genes were tightly correlated suggesting that these two biological processes are acting in synergy. Finally, the two copies of FMO gene reached their highest expression 3 days after infiltration suggesting that MdMYB52 is also involved in the regulation of these genes and by extension in the production of reactive oxygen species and systemic acquired resistance (as mentioned in the previous section). Finally, Cluster 3 did not display any transient expression pattern. The expression of genes belonging to this cluster was constantly increasing during the course of the experiment. The cluster includes genes coding for an ethylene responsive factor 15 (ERF15), trypsin inhibitor 1-like (TI1), xylem NAC domain 1 (XND1), a Casparian protein (CASP) and a laccase 14 (LAC14). ERF15 is a well-known stress responsive gene, ERF transcription factors are considered as central hubs in the regulation of hormonal cross-talk, as well as stress signaling during biotic and abiotic stress (Müller and Munné-Bosch, 2015). As mentioned above, XND1 is a negative regulator of the secondary cell wall biogenesis which includes lignin biosynthesis and it also mediates the PCD in the xylem (Zhao et al., 2010).

### Nicotiana leaves expressing MdMYB52 displayed an increase in lignin content

**Figure 5:**
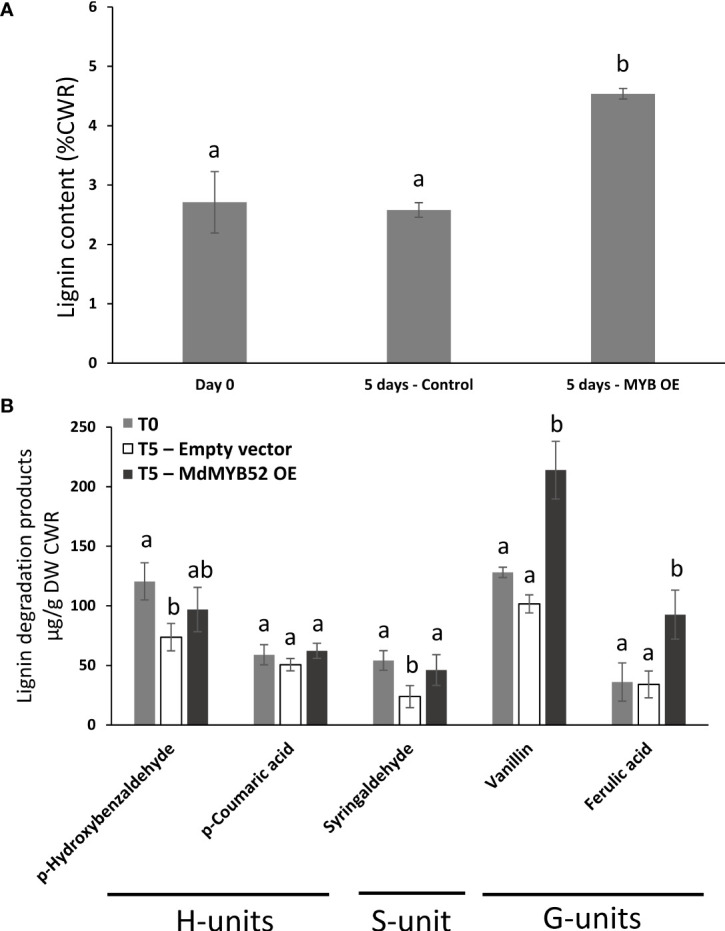
Total lignin content obtained using acetyl bromide methodology **(A)** and monomeric lignin composition obtained using nitrobenzene methodology **(B)**, in non-infiltrated leaves at T = 0 day (Day0), leaves infiltrated with P103 empty vector/pBIN61-p19 (Control) or p103::MD05G1011100/pBIN61-p19 (MdMYB52 OE) after 5 days. CWR = cell wall residue. Statistical analysis (n = 4) was performed from untransformed data using an ANOVA one-way followed by a Tukey’s *Post-Hoc* test. Letters display the significantly different groups.

In order to assess the role of MdMYB52 in the regulation of phenylpropanoid and lignin biosynthesis, we determined the total lignin content using an adapted protocol based on acetyl bromide in Nicotiana leaves expressing or not MdMYB52 (Behr et al., 2018). In leaves collected before infiltration (T=0 days) and control plants collected 5 days after infiltration with *A. tumefaciens* transformed with empty vector, the total lignin content remained stable with 2.71% (± 0.51) and 2.58% (± 0.12) of the cell wall residue, respectively (p-value=0.69) (**Figure 5A**). Inversely, leaves expressing MdMYB52 displayed a 75.9% increase in total lignin content in the cell wall residue when compared to control leaves at the same sampling date (4.53% ± 0.09%, p-value<0.0001). A deeper analysis was performed using a nitrobenzene oxidation methodology, in order to investigate the relative composition in this newly synthesized lignin. When comparing non-infiltrated leaves (T=0) to those expressing MdMYB52 or infiltrated with *A. tumefaciens* transformed with the empty vector after 5 days, only slight differences were observed for lignin degradation product corresponding to the H- et S-units (**Figure 5B**). A slight but significant decrease in *p*-hydroxybenzaldehyde and syringaldehyde oxidation products was observed between control leaves at T=0 days and Control at T=5 days, which might be due to the growth of the leaves during this period. Inversely, a significant increase in G-unit oxidation products was observed 5 days after MdMYB52 transient expression. The vanillin and ferulic acid displayed a 2.1- and 2.72-fold significant increase, respectively (**Figure 5B**). These results support the gene expression profiling data. The increased expression of genes associated with phenylpropanoid, monolignols and lignin biosynthetic pathways effectively triggered the synthesis of the lignin and, more particularly, the specific synthesis of G-unit lignin monomers. Regarding the expression of MdMYB52 in the suberized tissues, this specific increase in G-units including ferulic acid is highly relevant. Previous studies demonstrated that the so called lignin-like polyaromatic domain of suberin is enriched in G-units including ferulic acid and feruloyl-tyramine (Bernards, 2002; Graça, 2015; Woolfson et al., 2022). We can hypothesize that MdMYB52 has a role in the regulation of the synthesis of this specific G-units enriched polyaromatic domain.

### Histological analysis of tobacco leaves expressing MdMYB52 suggest a loss of integrity of the mesophyll parenchyma associated with secondary cell wall modification

In order to visualize histological differences in empty vector- and MdMYB52-agroinfiltrated leaves, cross-sections of the palisade/spongy parenchyma and central vein were prepared from tobacco plants for optical microscopy. FASGA was used to stain the phenolic hydroxyl groups of lignified tissues, then immunohistochemical staining with the monoclonal antibody LM10 was performed because of the specific affinity for xylan (unsubstituted). Observations of the xylem tissue did not show major differences between control and MdMYB52-agroinfiltrated leaves (**Supplementary Figures 4A, B**). The transient expression of MdMYB52 caused instead a loss in the integrity of the leaf palisade parenchyma (**Supplementary Figures 4C, D**). More specifically, the size of the intercellular spaces among the palisade cells increased (evidenced by the asterisks in **Supplementary Figure 4D**).

**Figure 6:**
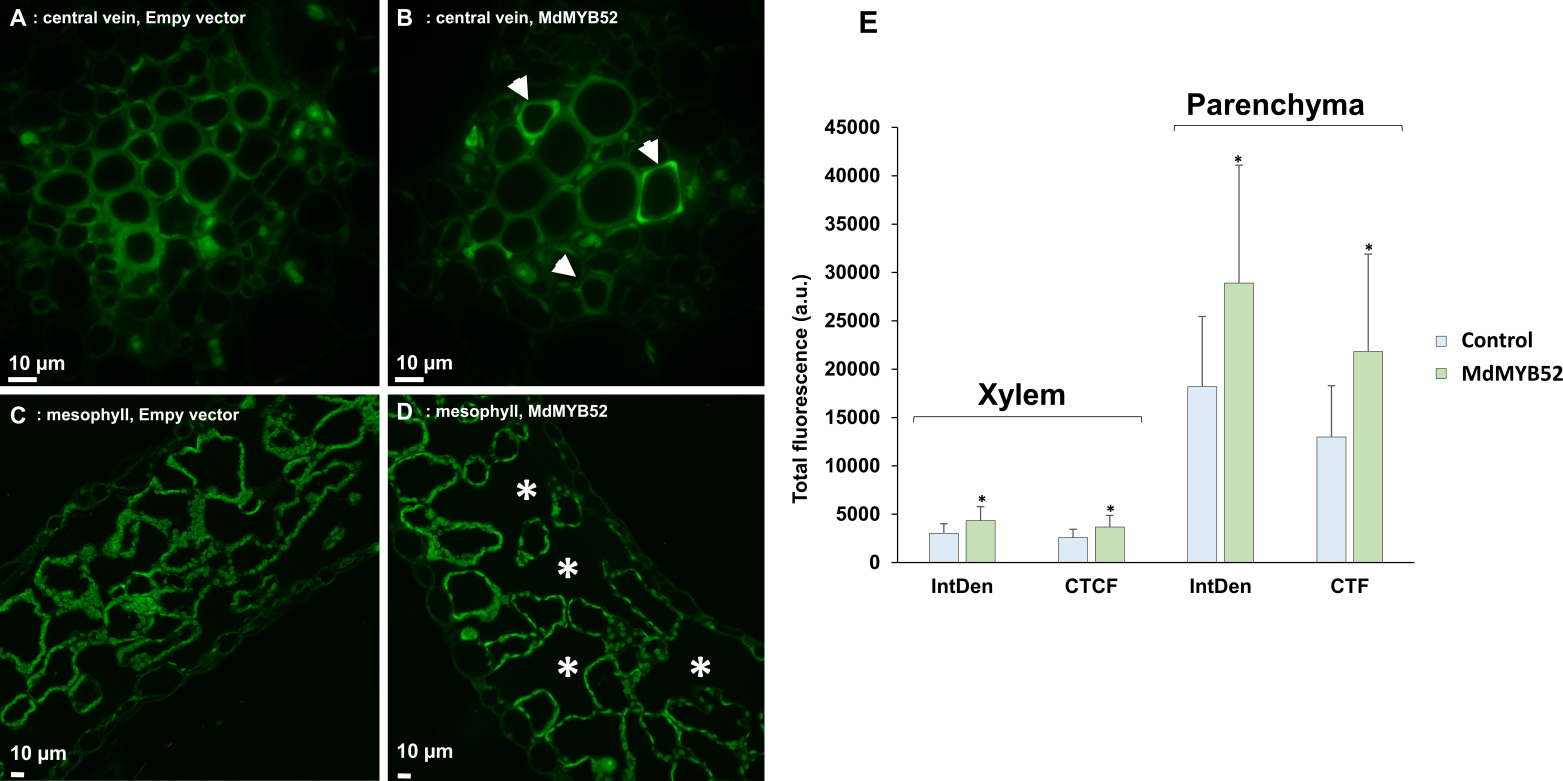
Thin sections of P103 empty vector/pBIN61-p19- **(A)** -central vein and **(C)** -mesophyll) and p103::MD05G1011100/pBIN61-p19-agroinfiltrated leaves **(B)** -central vein and **(D)** -mesophyll) incubated with the LM10 antibody. Arrows indicate cell wall areas with intense fluorescence and asterisks increased intercellular spaces. **(E)** total fluorescence detected in the xylem and parenchyma of P103 empty vector/pBIN61-p19- and p103::MD05G1011100/pBIN61-p19-agroinfiltrated leaves. * display significant satistical difference. The values were calculated through the software ImageJ and represented as integrated density (IntDen) and corrected total cell fluorescence (CTCF) in arbitrary units. Statistical values (n = 3): IntDen xylem T(38) = -2.750, p = 0.009, parenchyma T(38) = -3.204, p = 0.003; CTCF xylem T(38)=-3.319, p = 0.009, parenchyma T(38) = -3.518, p = 0.001.

The LM10 antibody shows the distribution of the hemicellulose xylan in secondary cell walls. It was reasoned that any induction of secondary cell wall formation in tobacco leaves agroinfiltrated with the MdMYB52 construct would result in an increased xylan signal. Differences were clearly detected in the xylem tissues of the central leaf vein (**Figures 6A, B**) where a stronger fluorescence was evident in the vessels of leaves expressing the transcription factor. The parenchyma showed only a slight increase in fluorescence (**Figures 6C, D**). However, the total fluorescence expressed both as IntDen and CTCF showed statistically significant differences both in the xylem and parenchyma tissues of leaves expressing MdMYB52 (**Figure 6E**). The log-transformed distribution of grey pixels confirms what observed with the LM10 antibody: a higher number of pixels with high grayscale intensity is present in the images taken from the parenchyma (**Supplementary Figures 5A, B**) and central vein (**Supplementary Figures 5C, D**) of MdMYB52-infiltrated leaves compared to the samples infiltrated with the empty vector.

## Discussion

The suberization of apple fruit skin, also called russeting, is a complex biological process, which recruits different metabolic pathways such as the lipid, sterol, phenylpropanoid and lignin metabolic pathways as well as cell wall modification processes (Legay et al., 2015; Falginella et al., 2021). These processes are tightly regulated at multiple levels to finely orchestrate the spatial deposition of suberin in the inner part of the cell wall. These last decades, the understanding of the biosynthesis of the aliphatic domain of suberin has been strongly improved. Major advances have been done regarding the biosynthesis of suberin building blocks, their export to the cell wall and, more recently, the final assembly of the aliphatic domain of suberin. However, the synthesis of the lignin-like polyphenolic domain or, even more, the alteration of the cell wall components, were less studied. Our previous transcriptomic studies showed that these two processes might be crucial for the anchoring of the aliphatic domain of suberin onto the cell wall (Legay et al., 2015; Legay et al., 2016). A similar alteration of the expression of genes associated with xyloglucan and xylan biosynthesis, as well as lignin biosynthesis was observed in both russeted apples and *N. benthamiana* leaves expressing the MdMYB93 master regulator, suggesting that these are fully integrated in the suberization program. Moreover, the regulation of the fate of peridermal suberized cells is still unclear. The russeted apple skin is made of dead suberized peridermal cells and the events and/or regulatory mechanisms leading to this cell death are still not fully deciphered and understood.

Several suberin synthesis transcriptional regulators have been discovered these last years, but only few of them have been deeply investigated for their role in the synthesis of the polyphenolic domain of suberin. In our previous work, we identified a MYB-domain transcription factor (MD05G1011100) whose expression profile displayed a high correlation with key suberin biosynthetic and regulatory genes (Legay et al., 2015; Legay et al., 2016; Legay et al., 2017). To better define its role and putative involvement in this biological process, we first performed a phylogenetic analysis based on protein sequence against a set of *A. thaliana* MYB transcription factors (**Figure 1**). This revealed that this transcriptional regulator is not clustered within the clade that includes already described suberin master regulators, such as MYB93, MYB92, MYB53, MYB41, MYB9 or MYB107 to name a few (Kosma et al., 2014; Lashbrooke et al., 2016; Legay et al., 2016; Shukla et al., 2021). MD05G1011100 is, in fact, clustered in a new clade, which includes MYB52, MYB54, MYB105, MYB117, MB110, MYB69, MYB56 and MYB89. It is noteworthy that, according to the clustering methodology, the obtained results varied slightly, whereas clade members did not change. One explanation for this is that, as observed for MdMYB93 (Legay et al., 2016), MD05G1011100 contains a disordered sequence, which is weakly conserved compared to the Arabidopsis orthologous gene and strongly impacts the results obtained in the phylogenetic analysis. Alternatively, using NCBI blastX against the *A. thaliana* database, the highest identity level (83.64%) was obtained against AtMYB52 with 31% coverage, the remaining part corresponding, again, to the intrinsically disordered region. Interestingly, AtMYB52 and other members of the same clade (MYB54, MYB69) were previously described as regulators of secondary cell wall biogenesis (Zhong et al., 2008), thus we first suspected that MdMYB52 might contribute to the cell wall modification/loosening observed in our previous transcriptomics studies (Legay et al., 2015; André et al., 2022).

The role of AtMYB52 in the regulation of the cell wall biogenesis was confirmed in other studies, but none of these provided any insights about a putative function in the suberization process in plants (Park et al., 2011; Xiao et al., 2021). Yet, MdMYB52 is specifically expressed in the suberized skin of apple and only weakly expressed in waxy tissues, suggesting a specific role of MdMYB52 in the suberization process (**Figure 2**). Its expression is also triggered upon MdMYB93 overexpression in *N. benthamiana* (Legay et al., 2016). In the suberized tissues, MdMYB52 displayed a high correlation with C4H and a slightly lower with GPAT5 and MdMYB93. As an example, in Canada Gris (**Figure 2C**), the couples MdMYB93/GPAT5, on one hand and MdMYB52/C4H, on the other hand did not display similar expression patterns during the course of the fruit growth. This suggests that MdMYB52 is not involved directly in the biosynthesis of the suberin aliphatic domain. Transcriptomic data obtained from *N. benthamiana* leaves expressing MdMYB52 confirmed this trend: no genes associated with the synthesis of the suberin aliphatic domain showed any altered expression (**Figure 3**). However, a significant increase of biological processes linked to the phenylpropanoid and lignin biosynthesis was observed, which is in agreement with previous studies (Zhong et al., 2008; Zhao et al., 2010). The analysis of the lignin content confirmed the results obtained from the whole gene expression study: overexpression of MdMYB52 significantly increased the total lignin content and particularly the biosynthesis of lignin G-units (**Figure 5**). This last result is particularly noteworthy as the proposed structural models state that the polyaromatic domain of suberin can be described as a lignin-like polymer enriched in guaiacyl lignin, as well as ferulic acid, a major component of suberin (Bernards, 2002; Graça, 2015; Woolfson et al., 2022). The increased expression of genes similar to the SAAP, LAC14 and tyramine N-feruloyltransferase further supports the hypothesis of a specific role of MdMYB52 in the regulation of the suberin polyaromatic domain biosynthesis. Laccase 14 has been previously described as a key factor in the deposition of guaiacyl lignin during the secondary cell wall biogenesis but, more recently, the increased expression of LAC14 has been associated with the occurrence of the suberization process in apple and poplar (Legay et al., 2015; Rains et al., 2018; André et al., 2022). The tyramine *N*-feruloyltransferase is involved in the synthesis of feruloyltyramine, which has been found in suberized periderms of solanaceous species such as tomato and potato, but also present in other species (Bernards et al., 1995; Bernards et al., 1999; Graça, 2015). The role of feruloyltyramine in the suberin polymer is still unclear, but a recent study showed that it is a crucial component of the ligno-suberin deposition in vascular cell walls, which leads to a reduction of the pathogen colonization in tomato (Kashyap et al., 2022). Finally, the potato SAAP has been associated with the suberin deposition in potato (Bernards et al., 1999). Interestingly, the potato SAAP displays the highest specificity for ferulic (*o*-methoxyphenol) substituted substrates, which supports the results obtained from the lignin monomer analysis. MdMYB52 seems to play a crucial role in the regulation of G-units-enriched lignin synthesis during the suberization process in apple fruit skin.

Furthermore, MdMYB52 seems to have a broader role as its overexpression was also associated with a modification of the cell wall through an enrichment by unsubstituted xylan and possibly a modification of xyloglucans by XETs and expansins. This global modification of the cell wall upon the suberization process was observed in our previous transcriptomic work. The alteration of a significant number cell wall modification genes was interpreted as a cell wall loosening process in russeted apple fruit skin (Legay et al., 2015; Legay et al., 2016; André et al., 2022). We further hypothesized that this increased expression of cell wall modification genes was involved in the remodeling of the cell wall to anchor the lignin-like/suberin polyphenolic domain and, by extension, the suberin aliphatic domain in the cell wall. In this respect, MdMYB52 seems to be a key factor in the regulation of this polyphenolic domain biosynthesis during the suberization process. However, regarding its gene expression pattern in russeted apple skin, we can speculate that it is not directly driven by the MdMYB93 master regulator as observed with genes responsible for the suberin aliphatic domain biosynthesis. Further promoter analysis studies might answer this question and reveal the regulatory interplay occurring between the lipid, phenylpropanoid pathway or the cell modification pathway, to name a few. Our previous transcriptomic work revealed a massive number of MYB, NAC, basic helix-loop-helix, which could be involved in the suberization pathway (Legay et al., 2015; Falginella et al., 2021; André et al., 2022). To date, only suberin master regulators have been identified but other downstream regulators such as MdMYB52, might be considered as key player of the whole suberin deposition in plants.

Finally, the overexpression of MdMYB52 in *N. benthamiana* leaves triggered a massive repression of genes belonging to photosynthesis and primary metabolism, concomitantly with an increased expression of stress responsive genes. Similar expression patterns were previously observed during intense osmotic and/or oxidative stresses (Rehem et al., 2012). A first explanation of this strong alteration of primary metabolism and photosynthesis-related genes could be that the “uncontrolled” increased expression of peroxidases led to an overaccumulation of reactive oxygen species in leaves expressing MdMYB52 and further triggered this stress response. QPCR analysis has highlighted a gene cluster, which displayed a continuous increase in expression during the course of the MdMYB52 overexpression (**Figure 3**). This include ERF15, Trypsin inhibitor, LAC14 and XND1. ERF15 is a positive regulator of ABA response under abiotic stresses including salinity and high osmolarity (Lee et al., 2015) and the trypsin inhibitor is also considered as a stress response/tolerance protein (Srinivasan et al., 2009). Interestingly, this putative stress response continuously increased during the course of the MdMYB52 transient expression study and continued this increase even 10 days after infiltration during which the expression of MdMYB52 and the above-mentioned lignin synthesis and cell wall modification genes were already abolished. This implies that the increased expression of these stress response genes might not be directly driven by MdMYB52 but resulted from the physiological effects of the induced downstream gene pool. Alternatively, we also observed an increased expression of genes which were previously associated with leaf senescence and PCD. Our first idea was that the observation was the result of this putative stress response. However, the gene expression analysis performed during the time-course kinetics of the MdMYB52 transient expression and downstream genes revealed that the senescence associated transcription factor WRKY53 (Miao et al., 2004) displayed the highest expression level only two days after infiltration, suggesting it was tightly regulated by MdMYB52. Altogether, these findings suggest that the stress phenotype, as well as the stress related gene expression pattern observed during MdMYB52 overexpression might not only be a direct consequence of the massive increased expression of peroxidases but might instead be due to a more global mechanism which leads the suberized cells into a PCD process. Further studies based on stable transformation with inducible promotor or Knock-Out strategy have to be carried out in order to assess this hypothesis; however, such phenomenon could partly explain the fate of suberized peridermal cells of the russeted apple fruit skin.

In conclusion, in the present study, we further elucidate the role of MdMYB52 during the suberization/russeting of apple fruits. MdMYB52 can be considered as a downstream or side-stream regulator compared to MdMYB93 and other suberin master regulators which is involved in the synthesis of the so-called suberin G-units enriched lignin-like/polyphenolic domain of suberin through the regulation of the genes involved in the phenylpropanoid and cell modification genes. The role of MdMYB52 in leaf senescence has still to be clarified, but the early induced expression of genes involved in leaf senescence suggests it might also have a broader role in the suberization process in apple.

## Data availability statement

The original contributions presented in the study are publicly available. This data can be found here: NCBI, GSE212828.

## Author contributions

SL, XX, GG, KS, and J-FH designed the experimental setup. XX, RB, SL, and GG performed the sampling. XX, SL, and GG performed the transcriptomic work (RNA extraction, QC control, RT-qPCR). XX and RB performed the microscopy work. XX, AL, and CG performed the analytical chemistry work, data extraction, metabolite identification. XX and SL performed the statistical analysis. XX, RB, SL and GG wrote the article. XX, RB, SL, KS, GG, and J-FH involved in the manuscript refinement. All authors contributed to the article and approved the submitted version.

## Funding

SL is grateful to the National Research Funds (FNR, Luxembourg) for funding (DANTE-FNR-CORE C18/SR/12700474).

## Acknowledgments

The authors acknowledge Aude Corvisy and Laurent Solinhac for their invaluable technical help.

## Conflict of interest

The authors declare that the research was conducted in the absence of any commercial or financial relationships that could be construed as a potential conflict of interest.

## Publisher’s note

All claims expressed in this article are solely those of the authors and do not necessarily represent those of their affiliated organizations, or those of the publisher, the editors and the reviewers. Any product that may be evaluated in this article, or claim that may be made by its manufacturer, is not guaranteed or endorsed by the publisher.

## References

[B1] Agarwal P. K. Agarwal P. Reddy M. K. Sopory S. K. (2006). Role of DREB transcription factors in abiotic and biotic stress tolerance in plants. Plant Cell Rep. 25, 1263–1274. doi: 10.1007/s00299-006-0204-8 16858552

[B2] André C. M. Guerriero G. Lateur M. Charton S. Leclercq C. C. Renaut J. . (2022). Identification of novel candidate genes involved in apple cuticle integrity and russeting-associated triterpene synthesis using metabolomic, proteomic, and transcriptomic data. Plants Basel Switz 11, 289. doi: 10.3390/plants11030289 PMC8838389 35161271

[B3] Anil Kumar S. Hima Kumari P. Shravan Kumar G. Mohanalatha C. Kavi Kishor P. B. (2015). Osmotin: A plant sentinel and a possible agonist of mammalian adiponectin. Front. Plant Sci. 6. doi: 10.3389/fpls.2015.00163 PMC4360817 25852715

[B4] Babu M. M. (2016). The contribution of intrinsically disordered regions to protein function, cellular complexity, and human disease. Biochem. Soc Trans. 44, 1185–1200. doi: 10.1042/BST20160172 27911701 PMC5095923

[B5] Baggerly K. A. Deng L. Morris J. S. Aldaz C. M. (2003). Differential expression in SAGE: Accounting for normal between-library variation. Bioinforma. Oxf. Engl. 19, 1477–1483. doi: 10.1093/bioinformatics/btg173 12912827

[B6] Bartsch M. Gobbato E. Bednarek P. Debey S. Schultze J. L. Bautor J. . (2006). Salicylic acid–independent ENHANCED DISEASE SUSCEPTIBILITY1 signaling in arabidopsis immunity and cell death is regulated by the monooxygenase FMO1 and the nudix hydrolase NUDT7. Plant Cell 18, 1038–1051. doi: 10.1105/tpc.105.039982 16531493 PMC1425861

[B7] Behr M. Legay S. Žižková E. Motyka V. Dobrev P. I. Hausman J.-F. . (2016). Studying secondary growth and bast fiber development: The hemp hypocotyl peeks behind the wall. Front. Plant Sci. 7. doi: 10.3389/fpls.2016.01733 PMC5114303 27917184

[B8] Behr M. Sergeant K. Leclercq C. C. Planchon S. Guignard C. Lenouvel A. . (2018). Insights into the molecular regulation of monolignol-derived product biosynthesis in the growing hemp hypocotyl. BMC Plant Biol. 18, 1. doi: 10.1186/s12870-017-1213-1 29291729 PMC5749015

[B9] Bernards M. A. (2002). Demystifying suberin. Can. J. Bot. 80, 227–240. doi: 10.1139/b02-017

[B10] Bernards M. A. Fleming W. D. Llewellyn D. B. Priefer R. Yang X. Sabatino A. . (1999). Biochemical characterization of the suberization-associated anionic peroxidase of potato. Plant Physiol. 121, 135–146. doi: 10.1104/pp.121.1.135 10482668 PMC59361

[B11] Bernards M. A. Lopez M. L. Zajicek J. Lewis N. G. (1995). Hydroxycinnamic acid-derived polymers constitute the polyaromatic domain of suberin. J. Biol. Chem. 270, 7382–7386. doi: 10.1074/jbc.270.13.7382 7706282

[B12] Bilgin D. D. Zavala J. A. Zhu J. Clough S. J. Ort D. R. DeLucia E. H. (2010). Biotic stress globally downregulates photosynthesis genes. Plant Cell Environ. 33, 1597–1613. doi: 10.1111/j.1365-3040.2010.02167.x 20444224

[B13] Bindea G. Mlecnik B. Hackl H. Charoentong P. Tosolini M. Kirilovsky A. . (2009). ClueGO: A cytoscape plug-in to decipher functionally grouped gene ontology and pathway annotation networks. Bioinformatics 25, 1091–1093. doi: 10.1093/bioinformatics/btp101 19237447 PMC2666812

[B14] Cohen H. Fedyuk V. Wang C. Wu S. Aharoni A. (2020). SUBERMAN regulates developmental suberization of the arabidopsis root endodermis. Plant J. Cell Mol. Biol. 102, 431–447. doi: 10.1111/tpj.14711 32027440

[B15] Czarnocka W. Fichman Y. Bernacki M. Różańska E. Sańko-Sawczenko I. Mittler R. . (2020). FMO1 is involved in excess light stress-induced signal transduction and cell death signaling. Cells 9, E2163. doi: 10.3390/cells9102163 32987853 PMC7600522

[B16] Daccord N. Celton J.-M. Linsmith G. Becker C. Choisne N. Schijlen E. . (2017). High-quality *de novo* assembly of the apple genome and methylome dynamics of early fruit development. Nat. Genet. 49, 1099–1106. doi: 10.1038/ng.3886 28581499

[B17] Domergue F. Kosma D. K. (2017). Occurrence and biosynthesis of alkyl hydroxycinnamates in plant lipid barriers. Plants 6, 25. doi: 10.3390/plants6030025 PMC5620581 28665304

[B18] Earley K. W. Haag J. R. Pontes O. Opper K. Juehne T. Song K. . (2006). Gateway-compatible vectors for plant functional genomics and proteomics. Plant J. Cell Mol. Biol. 45, 616–629. doi: 10.1111/j.1365-313X.2005.02617.x 16441352

[B19] Edgar R. C. (2004). MUSCLE: Multiple sequence alignment with high accuracy and high throughput. Nucleic Acids Res. 32, 1792–1797. doi: 10.1093/nar/gkh340 15034147 PMC390337

[B20] Evers D. Lefèvre I. Legay S. Lamoureux D. Hausman J.-F. Rosales R. O. G. . (2010). Identification of drought-responsive compounds in potato through a combined transcriptomic and targeted metabolite approach. J. Exp. Bot. 61, 2327–2343. doi: 10.1093/jxb/erq060 20406784

[B21] Falginella L. Andre C. M. Legay S. Lin-Wang K. Dare A. P. Deng C. . (2021). Differential regulation of triterpene biosynthesis induced by an early failure in cuticle formation in apple. Hortic. Res. 8, 1–15. doi: 10.1038/s41438-021-00511-4 33790248 PMC8012369

[B22] Faust M. Shear C. B. (1972). Russeting of apples, an interpretive review. Hortscience 7, 233–235. doi: 10.21273/HORTSCI.7.3.233

[B23] Franke R. Schreiber L. (2007). Suberin–a biopolyester forming apoplastic plant interfaces. Curr. Opin. Plant Biol. 10, 252–259. doi: 10.1016/j.pbi.2007.04.004 17434790

[B24] Fraser C. M. Chapple C. (2011). The phenylpropanoid pathway in arabidopsis. Arab. Book 9, e0152. doi: 10.1199/tab.0152 PMC3268504 22303276

[B25] Gall H. L. Philippe F. Domon J.-M. Gillet F. Pelloux J. Rayon C. (2015). Cell wall metabolism in response to abiotic stress. Plants 4, 112–166. doi: 10.3390/plants4010112 27135320 PMC4844334

[B26] Gasic K. Hernandez A. Korban S. S. (2004). RNA Extraction from different apple tissues rich in polyphenols and polysaccharides for cDNA library construction. Plant Mol. Biol. Rep. 22, 437–438. doi: 10.1007/BF02772687

[B27] Graça J. (2015). Suberin: the biopolyester at the frontier of plants. Front. Chem. 3. doi: 10.3389/fchem.2015.00062 PMC4626755 26579510

[B28] Hartig S. M. (2013). Basic image analysis and manipulation in ImageJ. Curr. Protoc. Mol. Biol. 102, 14.15.1–14.15.12. doi: 10.1002/0471142727.mb1415s102 23547012

[B29] Janusz G. Pawlik A. Świderska-Burek U. Polak J. Sulej J. Jarosz-Wilkołazka A. . (2020). Laccase properties, physiological functions, and evolution. Int. J. Mol. Sci. 21, 966. doi: 10.3390/ijms21030966 PMC7036934 32024019

[B30] Jung S. Lee T. Cheng C.-H. Buble K. Zheng P. Yu J. . (2019). 15 years of GDR: New data and functionality in the genome database for rosaceae. Nucleic Acids Res. 47, D1137–D1145. doi: 10.1093/nar/gky1000 30357347 PMC6324069

[B31] Kashyap A. Jiménez-Jiménez Á. L. Zhang W. Capellades M. Srinivasan S. Laromaine A. . (2022). Induced ligno-suberin vascular coating and tyramine-derived hydroxycinnamic acid amides restrict ralstonia solanacearum colonization in resistant tomato. New Phytol. 234, 1411–1429. doi: 10.1111/nph.17982 35152435

[B32] Kosma D. K. Murmu J. Razeq F. M. Santos P. Bourgault R. Molina I. . (2014). AtMYB41 activates ectopic suberin synthesis and assembly in multiple plant species and cell types. Plant J. Cell Mol. Biol. 80, 216–229. doi: 10.1111/tpj.12624 PMC4321041 25060192

[B33] Kumar S. Trivedi P. K. (2018). Glutathione s-transferases: Role in combating abiotic stresses including arsenic detoxification in plants. Front. Plant Sci. 9. doi: 10.3389/fpls.2018.00751 PMC5999759 29930563

[B34] Lashbrooke J. Cohen H. Levy-Samocha D. Tzfadia O. Panizel I. Zeisler V. . (2016). MYB107 and MYB9 homologs regulate suberin deposition in angiosperms. Plant Cell 28, 2097–2116. doi: 10.1105/tpc.16.00490 27604696 PMC5059810

[B35] Lee S. Lee S. Kim S. Y. (2015). AtERF15 is a positive regulator of ABA response. Plant Cell Rep. 34, 71–81. doi: 10.1007/s00299-014-1688-2 25253450

[B36] Legay S. Cocco E. André C. M. Guignard C. Hausman J.-F. Guerriero G. (2017). Differential lipid composition and gene expression in the semi-russeted “Cox orange pippin” apple variety. Front. Plant Sci. 8. doi: 10.3389/fpls.2017.01656 PMC5623121 29018466

[B37] Legay S. Guerriero G. André C. Guignard C. Cocco E. Charton S. . (2016). MdMyb93 is a regulator of suberin deposition in russeted apple fruit skins. New Phytol. 212, 977–991. doi: 10.1111/nph.14170 27716944

[B38] Legay S. Guerriero G. Deleruelle A. Lateur M. Evers D. André C. M. . (2015). Apple russeting as seen through the RNA-seq lens: Strong alterations in the exocarp cell wall. Plant Mol. Biol. 88, 21–40. doi: 10.1007/s11103-015-0303-4 25786603

[B39] Marques A. V. Pereira H. (2013). Lignin monomeric composition of corks from the barks of betula pendula, quercus suber and quercus cerris determined by py–GC–MS/FID. J. Anal. Appl. Pyrolysis 100, 88–94. doi: 10.1016/j.jaap.2012.12.001

[B40] Miao Y. Laun T. Zimmermann P. Zentgraf U. (2004). Targets of the WRKY53 transcription factor and its role during leaf senescence in arabidopsis. Plant Mol. Biol. 55, 853–867. doi: 10.1007/s11103-004-2142-6 15604721

[B41] Mittler R. Kim Y. Song L. Coutu J. Coutu A. Ciftci-Yilmaz S. . (2006). Gain- and loss-of-Function mutations in Zat10 enhance the tolerance of plants to abiotic stress. FEBS Lett. 580, 6537–6542. doi: 10.1016/j.febslet.2006.11.002 17112521 PMC1773020

[B42] Mortazavi A. Williams B. A. McCue K. Schaeffer L. Wold B. (2008). Mapping and quantifying mammalian transcriptomes by RNA-seq. Nat. Methods 5, 621–628. doi: 10.1038/nmeth.1226 18516045 PMC13303166

[B43] Müller M. Munné-Bosch S. (2015). Ethylene response factors: A key regulatory hub in hormone and stress Signaling1. Plant Physiol. 169, 32–41. doi: 10.1104/pp.15.00677 26103991 PMC4577411

[B44] Nakasugi K. Crowhurst R. Bally J. Waterhouse P. (2014). Combining transcriptome assemblies from multiple *De novo* assemblers in the allo-tetraploid plant nicotiana benthamiana. PloS One 9, e91776. doi: 10.1371/journal.pone.0091776 24614631 PMC3948916

[B45] Niu F. Cui X. Zhao P. Sun M. Yang B. Deyholos M. K. . (2020). WRKY42 transcription factor positively regulates leaf senescence through modulating SA and ROS synthesis in arabidopsis thaliana. Plant J. Cell Mol. Biol. 104, 171–184. doi: 10.1111/tpj.14914 32634860

[B46] Nouri M.-Z. Moumeni A. Komatsu S. (2015). Abiotic stresses: Insight into gene regulation and protein expression in photosynthetic pathways of plants. Int. J. Mol. Sci. 16, 20392–20416. doi: 10.3390/ijms160920392 26343644 PMC4613210

[B47] Papadopoulos J. S. Agarwala R. (2007). COBALT: constraint-based alignment tool for multiple protein sequences. Bioinformatics 23, 1073–1079. doi: 10.1093/bioinformatics/btm076 17332019

[B48] Park M. Y. Kang J. Kim S. Y. (2011). Overexpression of AtMYB52 confers ABA hypersensitivity and drought tolerance. Mol. Cells 31, 447–454. doi: 10.1007/s10059-011-0300-7 21399993 PMC3887605

[B49] Pascoal Neto C. Rocha J. Gil A. Cordeiro N. Esculcas A. P. Rocha S. . (1995). 13C solid-state nuclear magnetic resonance and Fourier transform infrared studies of the thermal decomposition of cork. Solid State Nucl. Magn. Reson. 4, 143–151. doi: 10.1016/0926-2040(94)00039-f 7773647

[B50] Qin S. Fan C. Li X. Li Y. Hu J. Li C. . (2020). LACCASE14 is required for the deposition of guaiacyl lignin and affects cell wall digestibility in poplar. Biotechnol. Biofuels 13, 197. doi: 10.1186/s13068-020-01843-4 33292432 PMC7713150

[B51] Qureshi M. K. Sujeeth N. Gechev T. S. Hille J. (2013). The zinc finger protein ZAT11 modulates paraquat-induced programmed cell death in arabidopsis thaliana. Acta Physiol. Plant 35, 1863–1871. doi: 10.1007/s11738-013-1224-y

[B52] Rains M. K. Gardiyehewa de Silva N. D. Molina I. (2018). Reconstructing the suberin pathway in poplar by chemical and transcriptomic analysis of bark tissues. Tree Physiol. 38, 340–361. doi: 10.1093/treephys/tpx060 28575526

[B53] Rehem B. C. Bertolde F. Z. Almeida A.-A. F. de (2012). “Regulation of gene expression in response to abiotic stress in plants”. in Cell metabolism (IntechOpen).

[B54] Robatzek S. Somssich I. E. (2002). Targets of AtWRKY6 regulation during plant senescence and pathogen defense. Genes Dev. 16, 1139–1149. doi: 10.1101/gad.222702 12000796 PMC186251

[B55] Shi D. Ren A. Tang X. Qi G. Xu Z. Chai G. . (2018). MYB52 negatively regulates pectin demethylesterification in seed coat Mucilage1. Plant Physiol. 176, 2737–2749. doi: 10.1104/pp.17.01771 29440562 PMC5884589

[B56] Shukla V. Han J.-P. Cléard F. Lefebvre-Legendre L. Gully K. Flis P. . (2021). Suberin plasticity to developmental and exogenous cues is regulated by a set of MYB transcription factors. Proc. Natl. Acad. Sci. U. S. A. 118, e2101730118. doi: 10.1073/pnas.2101730118 34551972 PMC8488582

[B57] Sievers F. Higgins D. G. (2014). Clustal omega, accurate alignment of very large numbers of sequences. Methods Mol. Biol. Clifton NJ 1079, 105–116. doi: 10.1007/978-1-62703-646-7_6 24170397

[B58] Skene D. S. (1982). The development of russet, rough russet and cracks on the fruit of the apple cox’s orange pippin during the course of the season. J. Hortic. Sci. 57, 165–174. doi: 10.1080/00221589.1982.11515037

[B59] Srinivasan T. Kumar K. R. R. Kirti P. B. (2009). Constitutive expression of a trypsin protease inhibitor confers multiple stress tolerance in transgenic tobacco. Plant Cell Physiol. 50, 541–553. doi: 10.1093/pcp/pcp014 19179349

[B60] Traore S. M. Zhao B. (2011). A novel gateway^®^-compatible binary vector allows direct selection of recombinant clones in agrobacterium tumefaciens. Plant Methods 7, 42. doi: 10.1186/1746-4811-7-42 22145613 PMC3265438

[B61] Velasco R. Zharkikh A. Affourtit J. Dhingra A. Cestaro A. Kalyanaraman A. . (2010). The genome of the domesticated apple (Malus × domestica borkh.). Nat. Genet. 42, 833–839. doi: 10.1038/ng.654 20802477

[B62] Vishwanath S. J. Delude C. Domergue F. Rowland O. (2015). Suberin: biosynthesis, regulation, and polymer assembly of a protective extracellular barrier. Plant Cell Rep. 34, 573–586. doi: 10.1007/s00299-014-1727-z 25504271

[B63] Woolfson K. N. Esfandiari M. Bernards M. A. (2022). Suberin biosynthesis, assembly, and regulation. Plants 11, 555. doi: 10.3390/plants11040555 35214889 PMC8875741

[B64] Xiao R. Zhang C. Guo X. Li H. Lu H. (2021). MYB transcription factors and its regulation in secondary cell wall formation and lignin biosynthesis during xylem development. Int. J. Mol. Sci. 22, 3560. doi: 10.3390/ijms22073560 33808132 PMC8037110

[B65] Yamamura M. Hattori T. Suzuki S. Shibata D. Umezawa T. (2010). Microscale alkaline nitrobenzene oxidation method for high-throughput determination of lignin aromatic components. Plant Biotechnol. 27, 305–310. doi: 10.5511/plantbiotechnology.27.305

[B66] Zentgraf U. Doll J. (2019). Arabidopsis WRKY53, a node of multi-layer regulation in the network of senescence. Plants 8, 578. doi: 10.3390/plants8120578 PMC6963213 31817659

[B67] Zhao C. Avci U. Grant E. H. Haigler C. H. Beers E. P. (2008). XND1, a member of the NAC domain family in arabidopsis thaliana, negatively regulates lignocellulose synthesis and programmed cell death in xylem. Plant J. Cell Mol. Biol. 53, 425–436. doi: 10.1111/j.1365-313X.2007.03350.x 18069942

[B68] Zhao Q. Wang H. Yin Y. Xu Y. Chen F. Dixon R. A. (2010). Syringyl lignin biosynthesis is directly regulated by a secondary cell wall master switch. Proc. Natl. Acad. Sci. U. S. A. 107, 14496–14501. doi: 10.1073/pnas.1009170107 20660755 PMC2922545

[B69] Zhong R. Lee C. Zhou J. McCarthy R. L. Ye Z.-H. (2008). A battery of transcription factors involved in the regulation of secondary cell wall biosynthesis in arabidopsis. Plant Cell 20, 2763–2782. doi: 10.1105/tpc.108.061325 18952777 PMC2590737

[B70] Zhou X. Jiang Z. Yu D. (2011). WRKY22 transcription factor mediates dark-induced leaf senescence in arabidopsis. Mol. Cells 31, 303–313. doi: 10.1007/s10059-011-0047-1 21359674 PMC3933965

