# Supplementary figures and images for: MdMYB52 regulates lignin biosynthesis upon the suberization process in apple
Source: Front Plant Sci. 2022 Oct 6;13:1039014. doi: 10.3389/fpls.2022.1039014 (PMC9583409; doi:10.3389/fpls.2022.1039014)

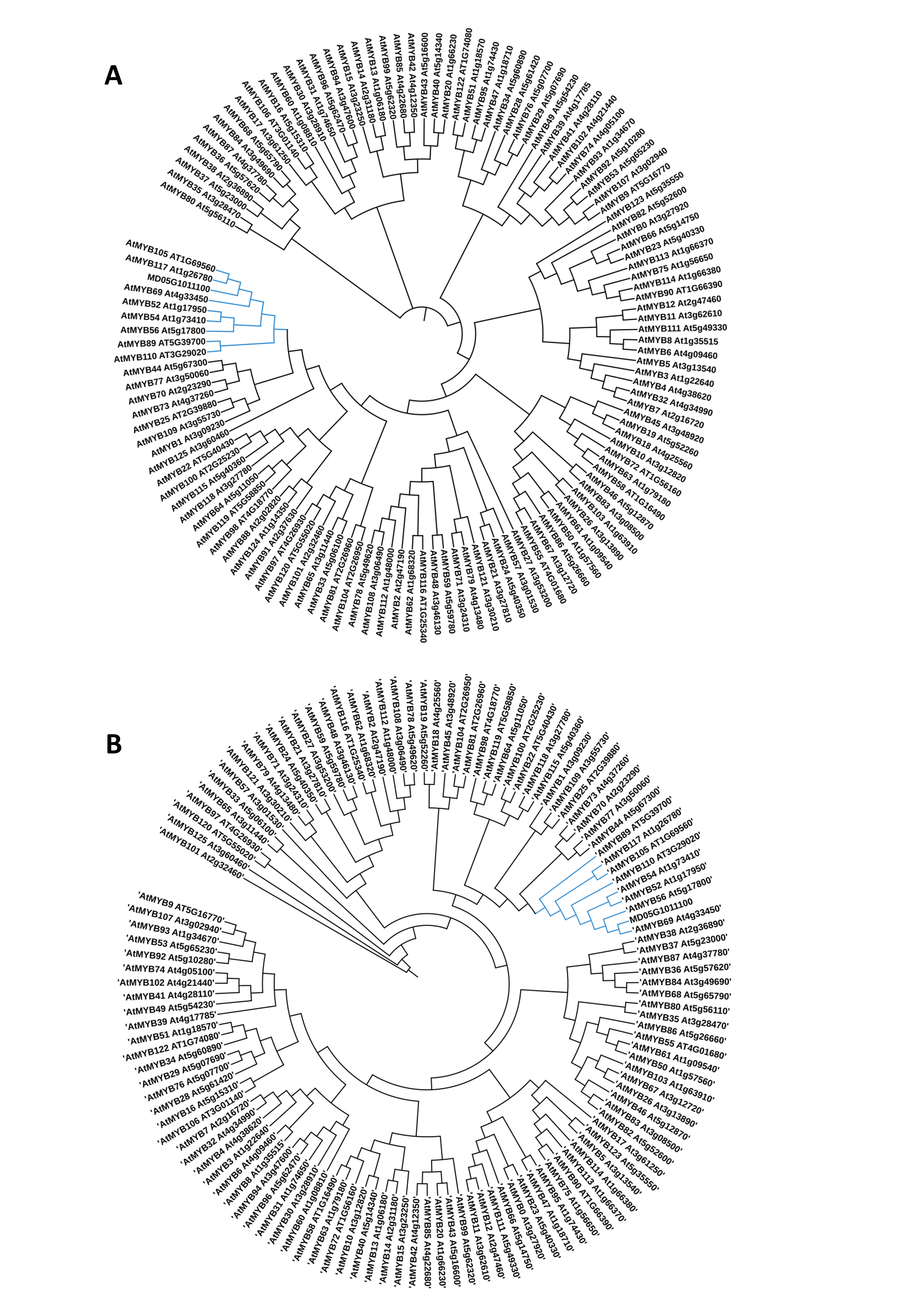

Supplement: Supplementary file 5 [file Image_1.tif]

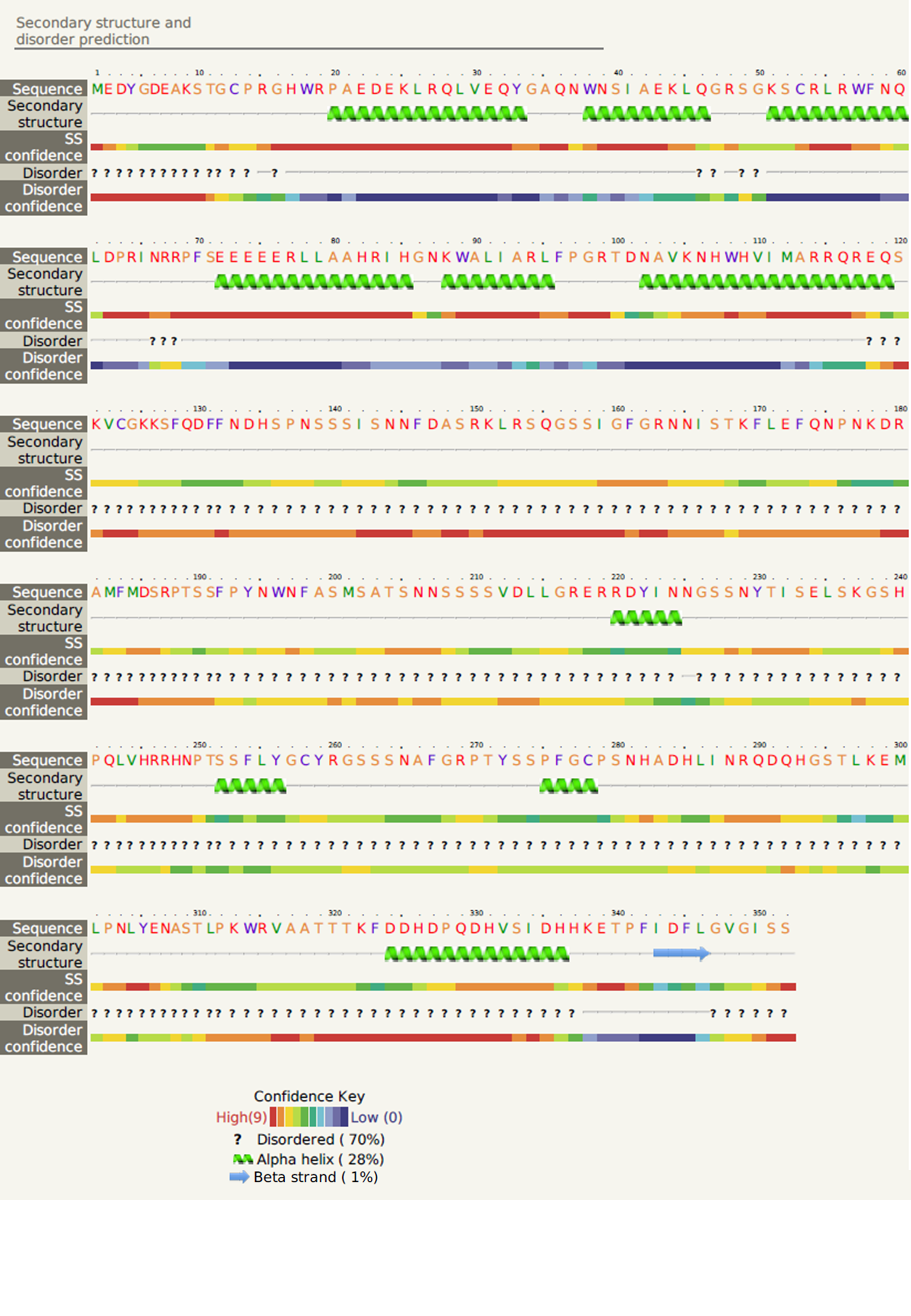

Supplement: Supplementary file 6 [file Image_2.tif]

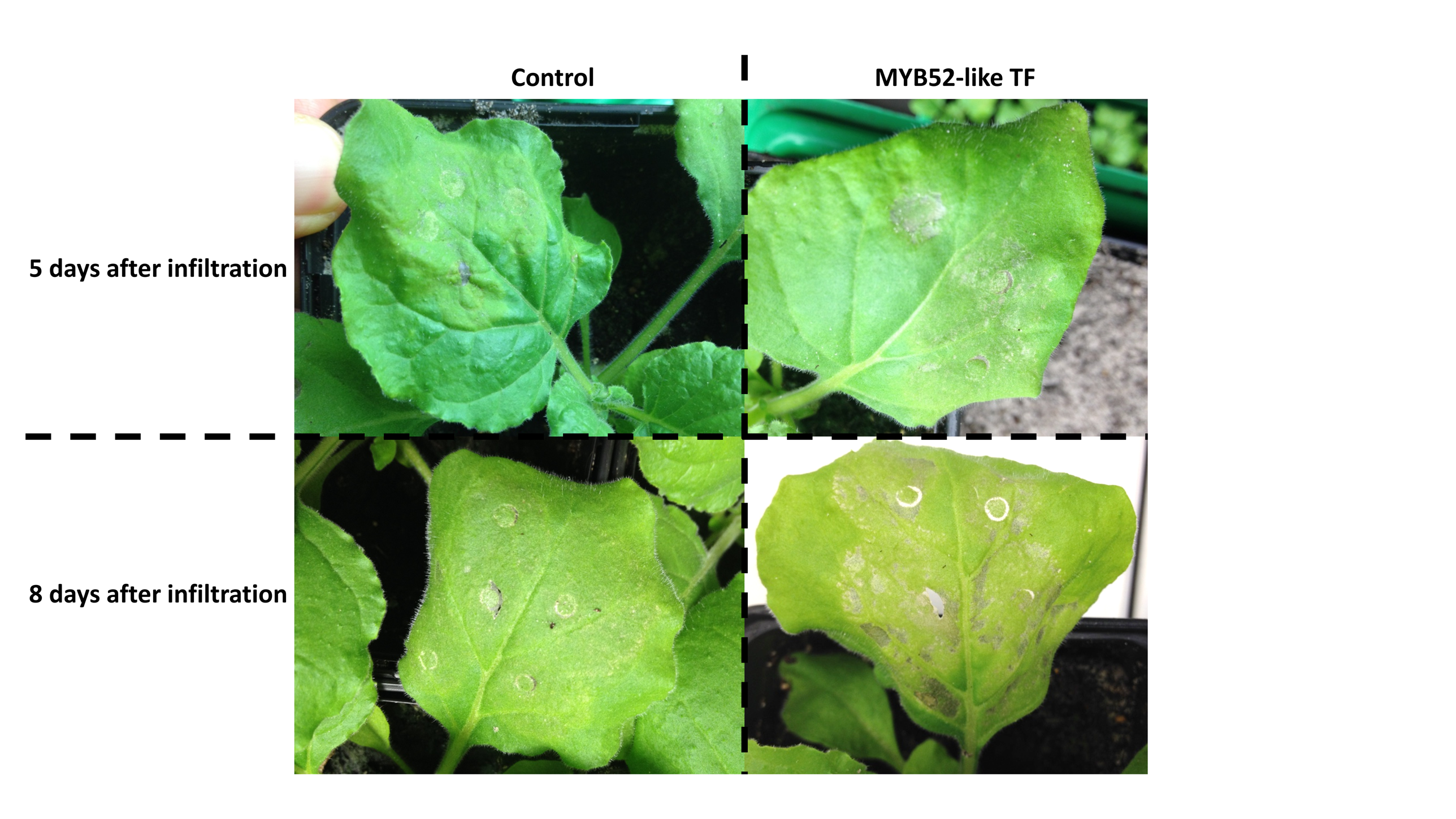

Supplement: Supplementary file 7 [file Image_3.tif]

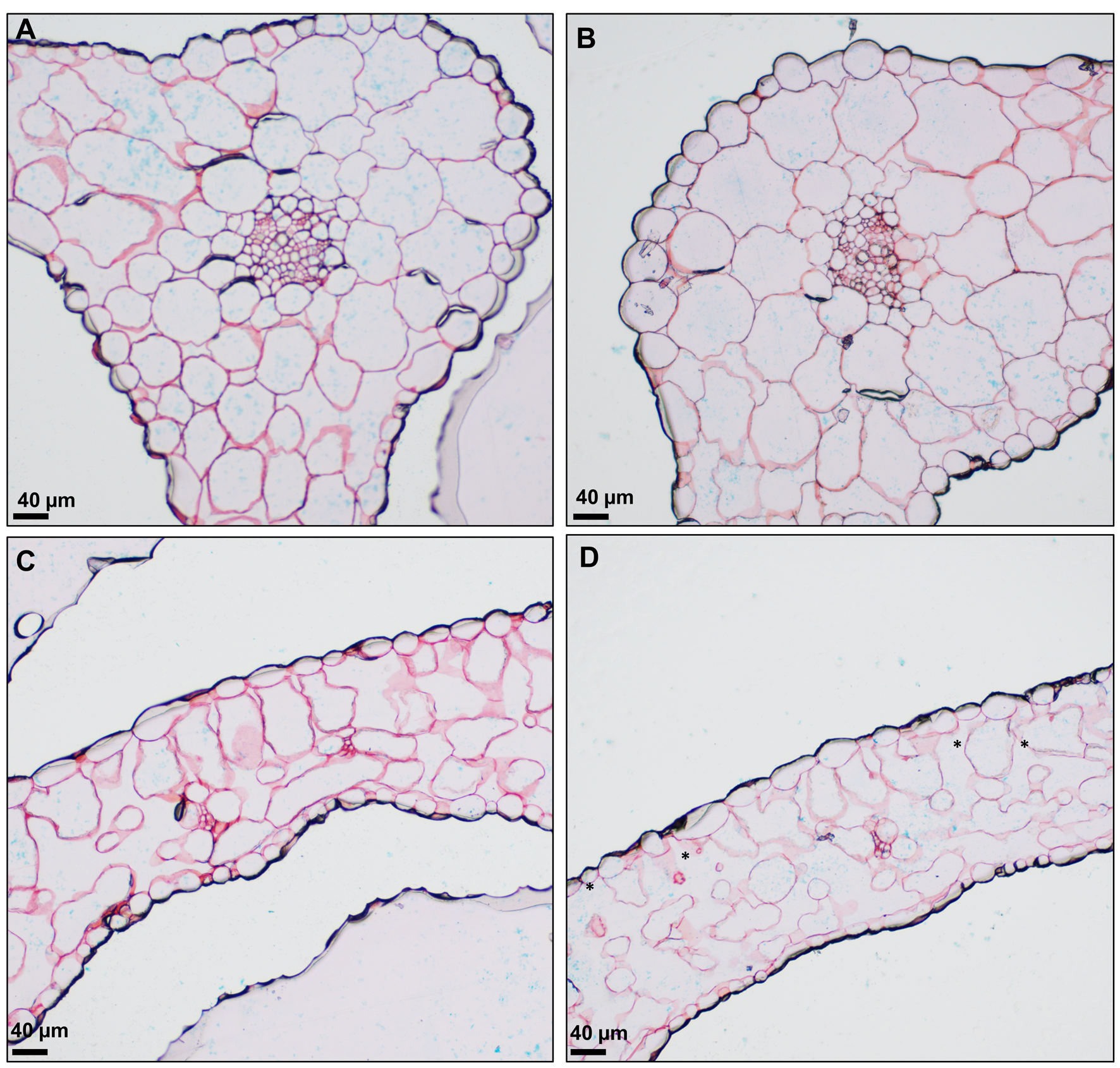

Supplement: Supplementary file 8 [file Image_4.tif]
